# How do you build a nectar spur? A transcriptomic comparison of nectar spur development in *Linaria vulgaris* and gibba development in *Antirrhinum majus*

**DOI:** 10.3389/fpls.2023.1190373

**Published:** 2023-06-23

**Authors:** Erin Cullen, Qi Wang, Beverley J. Glover

**Affiliations:** ^1^ Department of Plant Sciences, University of Cambridge, Cambridge, United Kingdom; ^2^ Department of Comparative Development and Genetics, Max Planck Institute for Plant Breeding Research, Köln, Germany

**Keywords:** *Antirrhinum majus*, gibba, *Linaria vulgaris*, nectar spur, RNA-seq, evo-devo

## Abstract

Nectar spurs (tubular outgrowths of floral organs) have long fascinated biologists. However, given that no model species possess nectar spurs, there is still much to learn about their development. In this study we combined morphological analysis with comparative transcriptomics to gain a global insight into the morphological and molecular basis of spur outgrowth in *Linaria*. Whole transcriptome sequencing was performed on two related species at three key developmental stages (identified by our morphological analysis), one with a spur (*Linaria vulgaris*), and one without a spur (*Antirrhinum majus*). A list of spur-specific genes was selected, on which we performed a gene enrichment analysis. Results from our RNA-seq analysis agreed with our morphological observations. We describe gene activity during spur development and provide a catalogue of spur-specific genes. Our list of spur-specific genes was enriched for genes connected to the plant hormones cytokinin, auxin and gibberellin. We present a global view of the genes involved in spur development in *L. vulgaris*, and define a suite of genes which are specific to spur development. This work provides candidate genes for spur outgrowth and development in *L. vulgaris* which can be investigated in future studies.

## 1 Introduction

**Figure 1:**
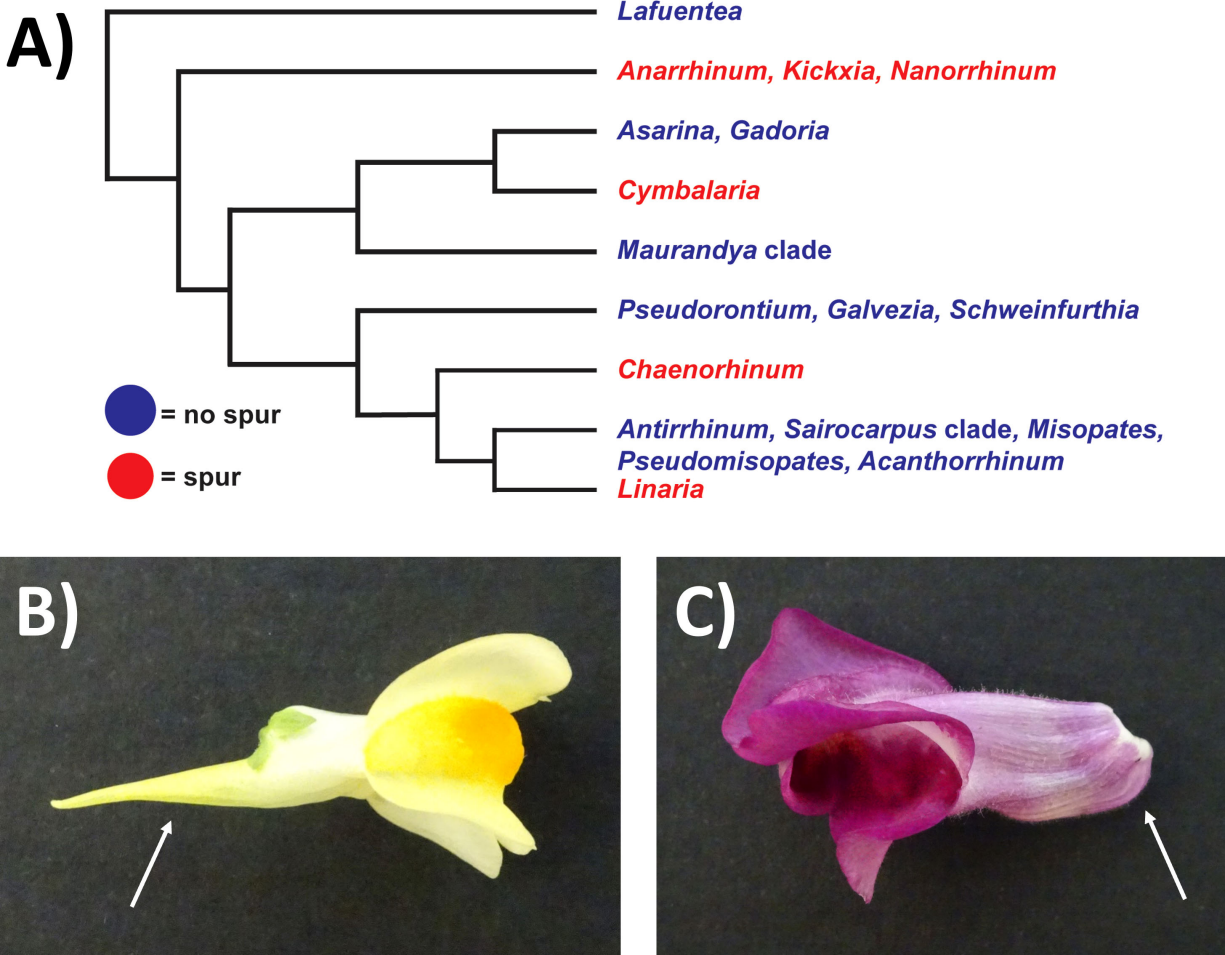
Schematic depicting phylogenetic relationships of the Antirrhineae and images of *Linaria vulgaris* and *Antirrhinum majus*. **(A)** Simplified phylogeny collapsed at the genus level, drawn from Fernández‐Mazuecos et al., 2019. Blue = a clade which does not possess a spur. Red = a clade which does possess a spur. **(B)** Photo of the mature flower of *L. vulgaris*. The location of the spur is indicated by the white arrow. **(C)** Photo of the mature flower of *A. majus*. The location of the gibba is indicated by the white arrow.

Biologists have been fascinated for centuries by nectar spurs (tubular outgrowths of the petal or sepal), which are thought to drive speciation (Hodges and Arnold, 1995; Fernández-Mazuecos and Glover, 2017). Darwin hypothesised that the extremely long nectar spur of the Orchid *Angraecum sesquipedale* allowed pollination by an equally long-tongued moth, and that the two species were locked into a ‘coevolutionary race’ (Whittall and Hodges, 2007; Micheneau et al., 2009). The evolution of nectar spurs across the *Aquilegia* (Ranunculales) and Antirrhineae (Lamiales) phylogenies was investigated by Whittall and Hodges (2007) and Fernández-Mazuecos et al. (2019) respectively. Both studies found that the ‘pollinator shift’ theory (where a plant evolves a longer spur in a short time frame in response to the longer feeding apparatus of a new pollinator) was more likely to explain the majority of spur-length evolution between different species than Darwin’s ‘coevolutionary race’ hypothesis. However, in the case of the Antirrhineae lineage in which nectar spurs evolved independently four times (see **Figure 1A**), additional factors such as the environment were suggested to play a role in spur-length evolution (Fernández-Mazuecos et al., 2019).

Nectar spurs have evolved multiple times, and occur in a phylogenetically diverse range of taxa, such as *Aquilegia*, *Linaria* and *Centranthus* (Dipsacales). Morphological analysis of nectar spur development has taken place in these three taxa. A key difference in spur morphology between the three groups is the location and means of nectar production. In *Aquilegia*, the nectary is located at the base of the spur (Yant et al., 2015); in *Centranthus* nectar is produced by trichomes within the spur; and in *Linaria*, the nectary is positioned on the gynoecium above the spur. The location and the number of spurs also differs between the species. *Aquilegia* possesses a spur on each petal, whereas in *Linaria* and *Centranthus* only one spur is present per flower (in *Linaria vulgaris* on the ventral petal, and in *Centranthus ruber* at the proximal end of the corolla). In three representative species from the above taxa (*L. vulgaris*, *C. ruber* and *A. coerulea*), a phase of initial cell division followed by cell expansion was observed in morphological analyses (Box et al., 2011; Mack and Davis, 2015; Yant et al., 2015). Intriguingly, the mechanism of spur length variation between species may differ in different systems. In *Aquilegia*, anisotropy (directed cell expansion) was found to be the cause of variation in spur length between different species (Puzey et al., 2012). However, in two sister *Linaria* species (one with a short spur and one with a long spur), cell division was found to be more important than cell length in generating differences in spur length (Cullen et al., 2018).

Nectar spurs develop late in floral development, and evidence suggests that they develop downstream of genes involved in the ‘ABC model’ and, where applicable, floral symmetry (Cubas et al., 1999; Kramer et al., 2007). In *Antirrhinum majus*, transposon insertions in the *KNOX* genes *HIRZINA* (*HIRZ*) and *INVAGINATA* (*INA*) were found to cause ectopic spur outgrowths (Golz et al., 2002). This work was followed up in the spurred species *L. vulgaris*. The expression of the orthologues of *HIRZ* and *INA* in *L. vulgaris* was discovered to be significantly higher in the ventral and dorsal petals than in non-floral organs, and ectopic expression in tobacco led to ectopic petal protrusions (Box et al., 2011). Therefore, these genes are good candidates for spur outgrowth in *L. vulgaris*. A potential master regulator of spur development in *Aquilegia*, *POPOVICH* (*POP*), was recently identified through genetic mapping (Ballerini et al., 2020). *POP* is a C2H2-type zinc finger transcription factor, which may be involved in the initiation of the spur through the regulation of mitosis (Ballerini et al., 2020). Another candidate gene for spur development in *A. coerulea* is *Teosinte Branched/Cycloidea/PCF 4* (*TCP4*), which was identified by RNAseq (Yant et al., 2015). The study above also suggested that auxin plays a key role in spur outgrowth in *Aquilegia*. When the *A. coerulea* orthologs of *ARF6* and *ARF8* were knocked down by VIGS, flowers with shorter spurs were observed (Zhang et al., 2020). While there has recently been significant progress on understanding the genes behind nectar spur development, much remains unknown. For example, which genes are involved in the initiation of the spur in *Linaria*? Which genes then promote the elongation of the emergent *Linaria* spur?

In this study, we compared *Linaria vulgaris* (**Figure 1B**), which possesses a spur, and *Antirrhinum majus* (**Figure 1C**), which possesses a gibba (a nectar containing pouch, thought to be the precursor to a spur). Both species are within the Antirrhineae, and diverged approximately 20 million years ago (Fernández-Mazuecos et al., 2019). These two species have been utilised in previous studies examining the basis of nectar spur development (Golz et al., 2002; Vincent and Coen, 2004; Box et al., 2011) and the genome sequence of *A. majus* was recently published (Li et al., 2019). Therefore, these two species are an excellent system for studying nectar spur and gibba development in a comparative context (Guzmán et al., 2015).

We aimed to gain insight into the morphological and molecular basis of spur outgrowth by:

1. Performing a detailed macro- and micromorphological analysis, to determine the time points for our RNAseq experiment.
2. Acquiring a global view of the genes involved in spur development.
3. Defining a list of candidate genes which are specific to the developing spur in *L. vulgaris*.

## 2 Material and methods

### 2.1 Plant material and growth conditions

The highly inbred *Antirrhinum majus* L. laboratory line 165E was used and *Linaria vulgaris* Mill. seeds were sourced from Emorsgate Seeds (Norfolk, UK). *A. majus* and *L. vulgaris* plants were grown in Levington’s M3 (UK) compost. Glasshouse conditions were maintained at 18-25°C, with 16-18 hr daylight, depending on the month plants were grown. Lights in the adjacent greenhouse were maintained at 24 hr lighting whilst plants were grown for material collection for transcriptome analysis, and therefore these plants may have received supplementary lighting.

### 2.2 Morphological analysis

A Dino-Lite digital microscope (Am400/AD4000 series, AM4113T(R4)) was used to take *in vivo* images of developing *L. vulgaris* or *A. majus* flowers. When flowers became too large to image consistently with the Dino-Lite digital microscope, images were taken on a mobile phone instead (HTC Desire 610), with a ruler for calibration. Five developing *L. vulgaris* spurs were imaged for 13 consecutive days. Spurs were measured from the calyx-corolla insertion to the tip using ImageJ (Schindelin et al., 2012), as described by Cullen et al. (2018). For *L. vulgaris*, the final data point (day 13) for spur length was not available for two of the replicates three days after anthesis as the flower had fallen off, and was not available for one replicate at day 10, or day 11. A lateral image of the spur was taken where possible. A different approach was necessary for the gibba measurements taken in *A. majus*, as the calyx obscured the gibba for much of development. Images of the entire flower were taken for 13 consecutive days and the length and width of the buds was measured (five replicates were taken from one plant, average length and width shown in **Table 1**). The average length and width of the bud was used to choose buds for imaging, and the calyx was removed before the gibba was imaged. There were five replicates at each time point aside from days eight and nine for which there were only three replicates and at days 10-12 where there were ten replicates.

**Table 1:** Average length and width of *A. majus* flower buds used in destructive timecourse measurements.

Day	Average length of *A. majus* bud (mm)	Average width of *A. majus* bud (mm)	Average length of *L. vulgaris* bud (mm)	Average width of *L. vulgaris* bud (mm)
-9	5.0	4.4	2.5	1.6
-8	5.9	4.6	3.0	1.8
-7	6.9	5.1	3.4	2.1
-6	9.0	5.9	3.9	2.3
-5	10.1	6.0	4.7	2.8
-4	12.2	6.3	7.2	3.0
-3	16.0	7.3	–	–
-2	18.6	7.9	–	–
-1	21.4	7.4	–	–
0	28.1	8.9	–	–
1	28.8	9.5	–	–
2	29.6	10.0	–	–
3	31.6	10.4	–	–

Equivalent measurements used for early and intermediate stages of *L. vulgaris* are also shown (data were not taken for days -3 to 3 for *L. vulgaris*). Anthesis occurs at day 0.

**Figure 2:**
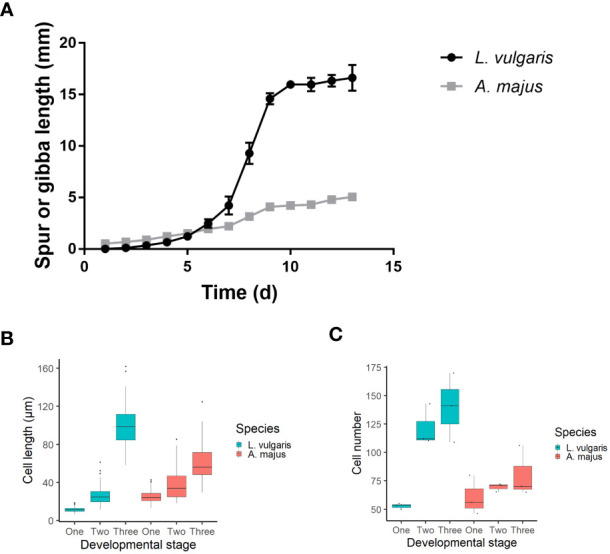
**(A)** A timecourse of spur and gibba growth. Anthesis occurred on day 10. Spur growth initiates later and has a higher growth rate than the gibba. The mean of five biological replicates is shown, +/- SE. **(B, C)** A comparison between cell length and number between the developing spur and gibba. The mean is shown +/-SE. **(B)** Cell length comparison between *A. majus* and *L. vulgaris*. Three biological replicates were taken for each species, with 30 replicates taken for each biological replicate. Cells were measured at the base of the spur/gibba. Data visualised using a boxplot; midpoint on boxplot indicates median, lines indicate the first and third quartiles. Outliers are indicated with dots. **(C)** Cell number comparison between the gibba of *A. majus* and the spur of *L. vulgaris*. Cells were counted along a single line from base to tip of the spur/gibba. Data visualised using a boxplot; midpoint on boxplot indicates median, lines indicate the first and third quartiles. Data from three replicates for each species are plotted on the boxplot.

Equivalent time points for microscopic analysis were determined by observing the spur or gibba growth curves over 13 days (**Figure 2A**). Material was taken approximately 6 days before anthesis (phase one), 3 days before anthesis (phase two) and from the mature flower (phase three) (**Table 2**). Three biological replicates were imaged for *L. vulgaris* and *A. majus* at the three developmental phases chosen. Material was dissected, then mounted on slides covered with double-sided sticky tape. Imaging was performed under standard settings with a digital microscope, VHX-5000 (KEYENCE, America), aside from the mature spur of *L. vulgaris*. Towards the tip of the spur in *L. vulgaris*, the epidermis becomes greatly striated, which obscures the cell boundaries and means that they cannot be accurately imaged using light microscopy. Therefore, for the mature spurs only, cryo-SEM was used to image the entire spur to enable an approximate estimate of cell number in the mature spur. Cryo-SEM was performed at the Sainsbury Laboratory Cambridge University. A Zeiss EVO HD15 coupled with a BackScatter Detector was used, and samples coated with 5nm platinum.

**Table 2:** Stages for cell size and number measurements.

Species	Stage one spur or gibba size (mm)	Stage two spur or gibba size (mm)	Mature spur or gibba
*L. vulgaris*	0.8	4	Open flower
*A. majus*	1.1	2	Open flower

### 2.3 Image and statistical analyses performed on morphological data

Image analysis was performed in ImageJ (Schindelin et al., 2012). The gibba of *A. majus* was defined for the purposes of this study as the region from the ventral side of the corolla-calyx insertion point until the point where the curved pouch became flat (**Figure S1**). To examine cell length and width, 30 epidermal cells were randomly chosen within the field of view. When examining cell length in the spur of *L. vulgaris* and the gibba of *A. majus*, only cells at the base of the spur or gibba were measured (**Figures S2A, B**). To count cell number, multiple images were taken along the length of the spur or gibba, and then merged in Adobe Photoshop so that epidermal cell number could be counted along the length of the spur or gibba (**Figures S2C, D**). A line was drawn along the length of the spur or gibba, and all cells dissected by this line were counted using the ‘Cell Counter’ ImageJ plug-in [as described in (Cullen et al., 2018)].

To determine whether there were differences in growth rate between *A. majus* and *L. vulgaris*, a grouped linear regression was used. It was necessary to determine where the steep increase in growth occurred in each species. For this the ‘segmented’ function in R was used to find two breakpoints on averaged data for each species (Muggeo, 2008; Lemoine, 2012). This divided up each species into three segments, and provided a gradient for each slope. The second segment gave the time points for the main growth phase for each species, and these time points were used in the grouped linear regression [method as described by (Cullen et al., 2018)]. To examine the initiation or end of spur or gibba growth, start (when a spur or gibba is first observed) and end (when the length of the spur or gibba no longer increases) of spur or gibba growth was noted. For *L. vulgaris*, initiation and end of spur growth was recorded for each of the five individual replicates and averaged. For *A. majus*, initiation and end of gibba growth was determined from averaged data as gibba measurements were destructive.

When analysing the cell length and number data, data were tested for normality and equality of variance (Dytham, 2010). Where data were parametric, a one-way ANOVA was used to test how developmental phase affected cell number in both *A. majus* and *L. vulgaris*. An independent samples t-test was used to compare cell number in the mature spur of *L. vulgaris* to the mature gibba of *A. majus*. Where data were not normally distributed or the variance was not equal, a non-parametric Kruskal-Wallis test and *post-hoc* Dunn test was used to test whether developmental phase had an effect on cell length in *A. majus* and *L. vulgaris*. A non-parametric Wilcoxon rank-sum test was used to compare cell length in the mature spur of *L. vulgaris* to the mature gibba of *A. majus*. The above statistical analyses were performed in R version 3.6.3.

### 2.4 Microdissection and RNA extraction

The time course data collected in this study was used to identify early, intermediate and late stages in *L. vulgaris* spur development. Tissue from the *A. majus* gibba was taken at the same time point as a control (see **Figure 2A**; **Table 1**). The three time points were as follows: pooled tissue eight or nine days prior to anthesis (early), pooled tissue four or five days prior to anthesis (intermediate) and two days prior to anthesis (late). Flowers were cut in half to separate the dorsal and ventral petals and the lobes of the petals were dissected off to remove any genes which may be involved in petal folding (**Figure S3**). Only petal tissue was retained. Material was collected from the two species, at the three different time points, and separated into the dorsal and ventral petals, thus a total of 12 tissue types (2x3x2). For each species, four individual plants were sampled, and each individual plant was a biological replicate. Therefore, there were four biological replicates for each of the 12 tissue types, making 48 samples in total.

For *A. majus*, the size of the flower buds to dissect was determined by taking the length and width data from the time course for *A. majus* (**Table 1**). For *L. vulgaris*, at the early and intermediate stages the length and width of buds was used to determine the size of the flower to take for dissection (also see **Table 1**); for the later stage, flowers were chosen that had a spur length of approximately 9.3 mm. The length and width of the flower bud was determined using digital callipers. Standard protocols for collecting tissue for RNA and DNA were followed. Plant material was collected at the same time each day, 12-3pm, and once collected material was frozen in liquid nitrogen and stored at -80°C. Material was placed into 2.0 ml Safe lock tubes (Fisher Scientific) with a 5 ml glass bead, and processed in a TissueLyser II (Qiagen) for 45 seconds to one minute at 30 Hz. RNA was processed and extracted with a Monarch Total RNA Miniprep kit (NEB). The workflow provided for Tough-to-Lyse Samples was followed, and the optional in-tube DNase I treatment was used to remove residual gDNA. RNA integrity was checked using a NanoDrop 2000 spectrophotometer (Thermo Fisher Scientific, Wilmington, USA).

### 2.5 Library preparation and Illumina sequencing

All library preparation and Illumina sequencing was performed by Novogene. Quantified libraries were aliquoted into Illumina sequencers Novaseq 6000 after pooling according to effective concentration and expected data volume. Paired-end 150bp sequencing was performed.

### 2.6 Transcriptome assembly and annotation

Transcriptome analysis was performed as shown in the workflow in **Figure S4**. Default parameters were used unless stated otherwise. Adaptors and low-quality bases were trimmed using cutadapt v.1.3 (Martin, 2011), and the quality of the data was checked using FastQC v.0.11.5 (*Babraham Bioinformatics - FastQC A Quality Control tool for High Throughput Sequence Data*). *De novo* transcriptome assembly was performed using Trinity v.2.8.4 (Haas et al., 2013); separate assemblies were created for *A. majus* and *L. vulgaris*. The quality of the transcriptome assemblies was evaluated as recommended in the Trinity documentation (Haas et al., 2013). First the percentage of reads mapped to the transcriptome assemblies was assessed using Bowtie2-2.3.4.2. Then the ExN50 statistic was calculated (**Figure S5**). In addition, representation of full-length reconstructed protein-coding genes in our assemblies was assessed against the *A. majus* reference proteome (http://bioinfo.sibs.ac.cn/Am/download_data_genome_v2/02.gene_predict/snapdragon_IGDBV1.pros.fasta.gz). Expression levels were quantified using Salmon v.1.0.0 (Patro et al., 2017). Corset v.1.07 was used to cluster the transcripts into genes and count the number of reads per gene with a distance threshold of 0.5 (Davidson and Oshlack, 2014). PCA analysis was performed with plotPCA function from DESeq2 package version 1.26.0, in R version 3.6.3 with default parameters. In the PCA plot, *A. majus* sample Am late dorsal biological replicate D(rD) was found not to cluster with the other dorsal samples of the same developmental stage, and therefore this outlier was not included in further analysis (**Figure S6**). Transdecoder v.5.5.0 was used to translate nucleotides into protein, where ORFs were identified with homology to known proteins (https://ftp.uniprot.org/pub/databases/uniprot/uniref/uniref90/uniref90.fasta.gz) via blast or to known protein domains via pfam searches (http://ftp.ebi.ac.uk/pub/databases/Pfam/releases/Pfam26.0/Pfam-A.hmm.gz). Transcripts were functionally annotated using Trinnotate v.3.1.1 (Pfam, UniProt, eggnog, and GeneOntology) (Bryant et al., 2017). Gene annotation shown on graphs was derived from the top BlastX hit to Uniprot (https://ftp.uniprot.org/pub/databases/uniprot/previous_releases/release-2019_04/knowledgebase/uniprot_sprot-only2019_04.tar.gz). To investigate cyclin expression patterns across the different developmental stages, cyclin genes were defined as genes annotated with the cyclin ‘N-terminal’ domain (PF00134.23) or ‘C-terminal’ domain (PF02984.19) by trinotate.

### 2.7 Orthology identification

To identify orthologs between *A. majus* and *L. vulgaris*, Orthofinder v.2.2.7 was used with the following species included in the analysis (Emms and Kelly, 2015): *L. vulgaris*, *A. majus*, *Arabidopsis thaliana*, *Solanum lycopersicum*, *S. tuberosum* and *Erythranthe guttata*. The full transcriptome assemblies of *L. vulgaris* and *A. majus* were also included in Orthofinder analysis. The annotated protein sequences of the other species were downloaded from publicly available sources: the *A. thaliana* protein sequences were extracted from (https://ftp.uniprot.org/pub/databases/uniprot/previous_releases/release-2019_04/knowledgebase/uniprot_sprot-only2019_04.tar.gz) using the keyword for species name ARATH, *S. lycopersicum* (ftp://ftp.solgenomics.net/tomato_genome/annotation/ITAG3.2_release/ITAG3.2_proteins.fasta), *S. tuberosum* (ftp://ftp.ensemblgenomes.org/pub/plants/release-44/fasta/solanum_tuberosum/pep/Solanum_tuberosum.SolTub_3.0.pep.all.fa.gz), *A. majus* (http://bioinfo.sibs.ac.cn/Am/download_data_genome_v2/02.gene_predict/snapdragon_IGDBV1.pros.fasta.gz) and *E. guttata* (ftp://ftp.ncbi.nih.gov/genomes/Erythranthe_guttata/protein/protein.fa.gz).

### 2.8 Supervised clustering of genes with similar expression patterns

To gain a global view of gene expression dynamics during spur and gibba development, supervised clustering was used to group differentially expressed (DE) genes according to their expression patterns. For *L. vulgaris*, comparisons were performed between the following tissue types to assess whether a gene was up-regulated, down-regulated, or not statistically significantly differentially expressed (NSD) comparing: (1) the ventral vs dorsal petal of *L. vulgaris* at each developmental stage (at an early, intermediate or late stage), (2) the ventral petal of *L. vulgaris* between each pair of developmental stages (intermediate vs. early, late vs. intermediate, late vs. early), (3) and the dorsal petal of *L. vulgaris* between each pair of developmental stages (intermediate vs. early, late vs. intermediate, late vs. early). The three sets of comparisons (1-3) were each summarised to identify the trend (up, down or NSD), which was then used to cluster the genes (**Table 3**). To summarise the trend within the set of comparisons for set (2) or (3), if a gene was upregulated in at least one pair of comparisons (intermediate vs. early, late vs. intermediate, late vs. early) and NSD in the others, it was summarised as ‘up’; if a gene was downregulated in at least one pair of comparisons (intermediate vs. early, late vs. intermediate, late vs. early) and NSD in the rest, it was summarised as ‘down’; if the differential expression was NSD in all comparisons within the set, it was summarised as not significantly differentially expressed, i.e., ‘NSD’. Any genes which were not classified into one of these three categories were assigned to cluster 7 (‘didn’t follow a consistent trend ‘) e.g. genes whose expression patterns went up and down across developmental stages, or went up in ventral across stages but down in dorsal, in at least one of comparison (1-3) (**Table 3**). For *A. majus* genes, the same clustering was performed.

**Table 3:** Details of how the seven clusters were assigned in *L. vulgaris* and *A. majus*.

Assigned cluster	(1) Difference between Ventral vs Dorsal	(2) Trend in Ventral over time	(3) Trend in Dorsal over time
1	Higher in ventral	Up/Nsd*	Up/Nsd*
2	Higher in ventral	Down/Nsd*	Down/Nsd*
3	Higher in ventral	Nsd	Nsd
4	Higher in dorsal	Up/Nsd^	Up/Nsd^
5	Higher in dorsal	Down/Nsd^	Down/Nsd^
6	Higher in dorsal	Nsd	Nsd
7	All remaining genes		

* if both (2) and (3) are Nsd, then the gene belongs to Cluster 3.

^ if both (4) and (5) are Nsd, then the gene belongs to Cluster 6.

The seven clusters which are found in the data are presented. Up = a log2 fold change > 1 and adjusted p-value < 0.1; down = log2 fold change < (-1) and adjusted p-value < 0.1; nsd, Not statistically significantly differentially expressed.

### 2.9 Identifying spur-specific genes

When comparing gene expression between tissues of the same species, the R package DESeq2 v.1.22.2 (Love et al., 2014) was used to call differentially expressed genes. Differential expression (including up and down regulation) was defined as false discovery rate (FDR) alpha less than 0.1 and log2 (log with base 2) fold change (LFC) greater than 1.0. When comparing gene expression between tissues of different species, a t-test of log2 transcripts per million (TPM) (Wagner et al., 2012; Martin and Fraser, 2018) was used with cut-off p-values less than 0.001 and LFC greater than 1.0. The TPM distributions were more similar between assemblies generated by trinity than between the assemblies and the reference transcriptome. Therefore, for comparing gene expression between tissues of different species, the TPM values based on the trinity assemblies were used. Sample Lv_Late_D_rD was excluded from TPM-based comparisons because of its high number of total reads, resulting in distortion after the TPM normalization. In TPM comparisons, when log2 (TPM) was less than -2, the gene was treated as not expressed in that sample.

To identify a ‘spur-specific’ set of genes, the following strategy was used. A gene was defined as a ‘spur aid’ gene if its expression in *L. vulgaris* ventral was the highest among the expression levels in the four relevant tissues of a developmental stage (i.e., *L. vulgaris* ventral, *L. vulgaris* dorsal, *A. majus* ventral and *A. majus* dorsal). To be specific, a ‘spur aid’ gene needed to meet all of the following three criteria in the developmental stage: (1) A statistically significant increase in its expression level in *L. vulgaris* ventral compared to that in *L. vulgaris* dorsal; (2) A statistically significant increase in its expression level in *L. vulgaris* ventral tissues compared with the expression level of this gene’s *A. majus* ortholog in *A. majus* ventral tissues; (3) The mean log2 TPM value in *L. vulgaris* ventral was at least 1.0 higher than this value in *A. majus* dorsal. Alternatively, a gene was defined as a ‘spur suppressor’ gene if it met all of these criteria in least one of the three developmental stages: (1) A statistically significant decrease in its expression level in *L. vulgaris* ventral compared to that in *L. vulgaris* dorsal; (2) A statistically significant decrease in its expression in *L. vulgaris* ventral tissues compared with the expression level of this gene’s *A. majus* ortholog in *A. majus* ventral tissues; (3) The mean log2 TPM value in *L. vulgaris* ventral was at least 1.0 lower than that in *A. majus* dorsal. The limitation of this method is that it can only evaluate whether a gene in *L. vulgaris* is ‘spur-specific’, where the gene has only one orthologous gene identified in *A. majus* by Orthofinder. When multiple orthologues in *A. majus* were identified for one gene (meaning that there was not enough information to identify the true orthologue in *A. majus*), this gene was not further considered as ‘spur-specific’ in *L. vulgaris*. If the *L. vulgaris* gene did not have an ortholog identified (see method section Orthology Identification) in the assembled *A. majus* transcriptome, then this gene was likely to be absent or lowly expressed in *A. majus* tissues included in this study. In this case, this gene only had to meet criterion (1) for both ‘spur aid’ and ‘spur suppressor’ genes. Heatmaps were drawn with ComplexHeatmap version 2.2.0 (Gu et al., 2016).

R (version 3.6.3) package TopGO (version 2.38.1) was applied to identify GO terms enriched among the *L. vulgaris* spur genes compared with the background, i.e. all expressed genes in either the dorsal or ventral part. A GO term is considered enriched if its p-value is lower than 0.005 and includes at least 5 annotated background genes. All genes were annotated to GO terms through top blastx hits among Uniprot proteins.

## 3 Results

### 3.1 A framework to inform transcriptome design

The spurred species *L. vulgaris* (**Figure 1B**), was compared to the related *A. majus*, a species which possesses a gibba (a nectar-containing pouch) (**Figure 1C**). Time courses were taken over 13 days to determine whether there was a difference in the growth duration and growth rate between the gibba of *A. majus* and the spur of *L. vulgaris* (anthesis occurred at day 10, **Figure 2A**). Spur growth initiated an average of eight days prior to anthesis (**Figure S7A**), and there was a steep increase in spur growth three days before anthesis (see **Figure 2A** and **Table 4** for a comparison of gradients of the slope). Spur growth levelled off when the flower opened. Gibba growth shows a different trend. At nine days prior to anthesis, there is already a very small gibba present (**Figure S7B**). Rather than a steep increase in gibba growth prior to anthesis, there is a gradual and steady increase in gibba size. A grouped linear regression comparing the growth period in *L. vulgaris* and *A. majus* determined by the segmented function (**Table 4**) showed that there was a significant difference in growth with time (p < 0.01). There was also a significant difference in growth between the two species (p < 0.001), and an interaction between species and time (p < 0.001).

**Table 4:** Table showing the date of average initiation and end of spur or gibba growth, plus the breakpoint in growth rate predicted by the segmented package.

Species	Average initiation of spur or gibba	Average end of spur or gibba growth	Day segmented function identified	Gradient
*A. majus*	1	13	7-9	1.0
*L. vulgaris*	2	12	7-9	5.2

The gradient of this section of the slope is also shown.

### 3.2 Cell number and cell length is higher in the *L. vulgaris* spur than the *A. majus* gibba

The length of the cells at the base of the structure were examined in both species at initiating (phase 1, approximately 6 days before anthesis) and elongating (phase 2, approximately 3 days before anthesis) phases of development and in the mature spur or gibba (phase 3) (see **Figures S2A, B**, for morphological description see **Table 2**). Cell length increases with time in both species; however they follow different trends (**Figure 2B**). In *L. vulgaris*, mean cell length increases from 11.5 μm at phase one to 26.3 μm at phase two. Cell length then rapidly increases from phase two to 98.7 μm in phase 3. There was a highly significant difference in cell length between the developmental phases (*X^2^ = *234.44; d.f. 2; p < 0.001), which a *posthoc* Dunn test indicated was significant between each developmental phase (p < 0.001). Conversely, in *A. majus*, cell length gradually increases from phase 1 to phase 3. There was also a significant difference in cell length between the developmental phases (*X^2^ = *158.59; d.f. 2; p < 0.001), which was also found to be significant at every developmental phase (p < 0.001) by a *posthoc* Dunn test. There was a significant difference in cell length between the phase 3 spur and gibba of the two species (*W* = 674; p < 0.001). Cell number (for definition see **Figures S2C, D**) in *L. vulgaris* increased from 52.7 to 121.7 from phase one to phase two and then had a small increase to approximately 140.0 in the phase 3 spur (**Figure 2C**). There was a significant difference in cell number between the different developmental phases in *L. vulgaris* (*F_2,6_ = *14.92; p < 0.01). A *posthoc* Tukey’s HSD test revealed that there was a significant difference between phase one and phase 3 (p < 0.01) and phase one and phase two (p < 0.05); however, there was no significant difference in cell number between phase two and phase 3 (p > 0.05). In contrast, in *A. majus*, there were small and steady increases in cell number throughout development. These were not found to be significantly different (*F* = 1.07; d.f. 2; p > 0.05). The ratio between the mean number of cells in the *L. vulgaris* spur and the *A. majus* gibba (the log fold change) was a factor of 1.7 higher in the mature spur than the gibba. This difference was below the statistical significance threshold between the two species, 0.05 (p = 0.052), perhaps due to a small sample size (t = -2.73; p > 0.05).

### 3.3 Results from *de novo* transcriptome assembly reflect our morphological analysis

**Figure 3:**
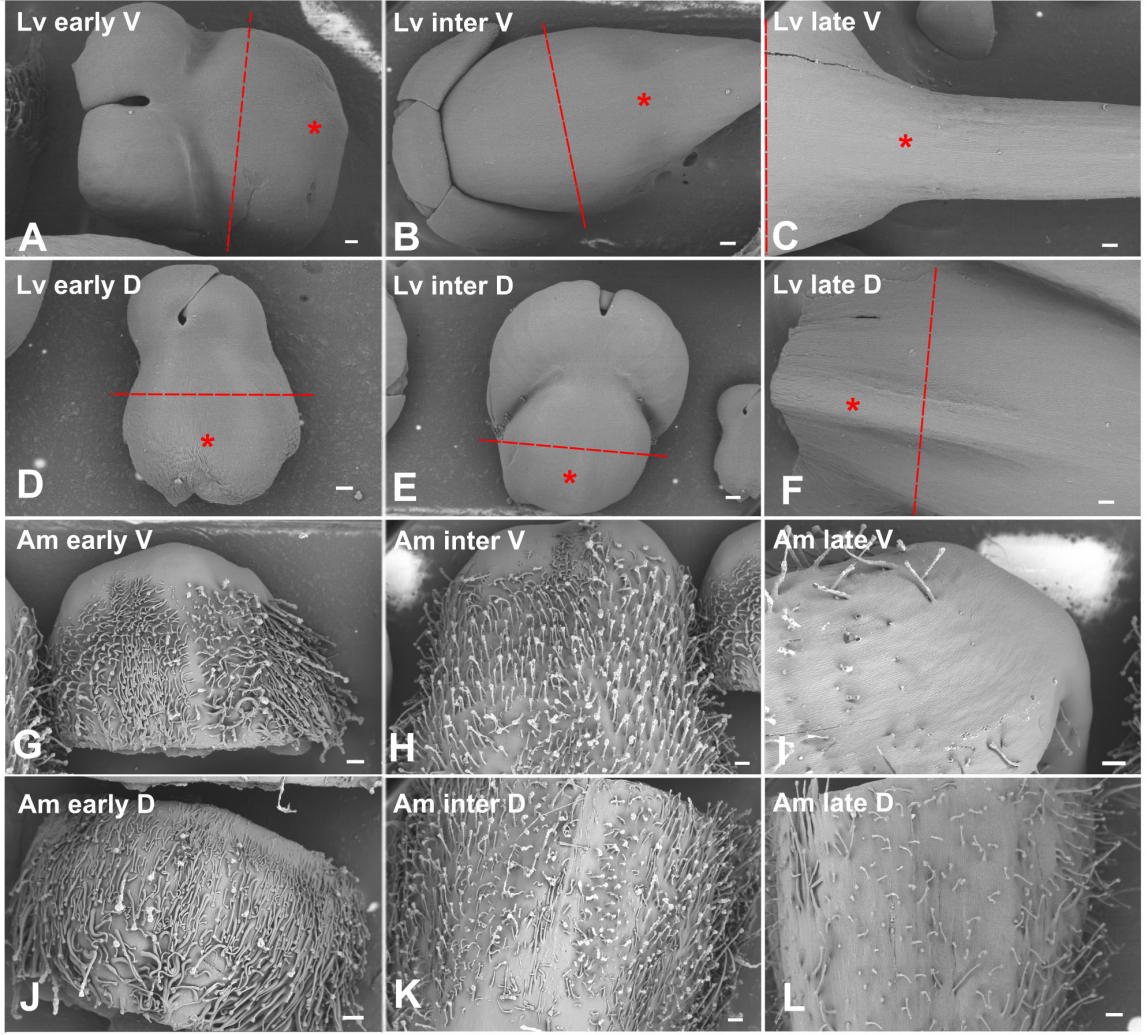
Stages of *L. vulgaris* and *A. majus* used for RNAseq, imaged with cryo Scanning Electron Microscopy. Red dashed line indicates where petal lobes were dissected off in *L. vulgaris* and the asterisk designates where material was taken for RNAseq after dissection. All material shown in *A. majus* images was processed for RNAseq. Scale bars represent 200 μm, aside from **(A, D)** where scale bars represent 100 μm. Lv, L. vulgaris; Am, A. majus; early, early stage; inter,intermediate stage; late, late stage; V, ventral; D, dorsal, **(A-F)** *L. vulgaris.* **(A-C)** Ventral petal. **(D-F)** Dorsal petal. **(A, D)** Early in spur development (eight or nine days before anthesis). **(B, E)** An intermediate phase in spur development (four or five days before anthesis). **(C, F)** A late stage in spur development (two days before anthesis). **(G-L)** *A. majus*. **(G-I)** Ventral petal. **(J-L)** Dorsal petal. **(G, J)** Early in gibba development (eight or nine days before anthesis). **(H, K)** An intermediate phase in gibba development (four or five days before anthesis). **(I, L)** A late stage in gibba development (two days before anthesis).

Informed by our morphological analysis, we chose an early, intermediate and late stage in spur and gibba development for our RNAseq experiment (this corresponds to eight or nine, four or five or two days before anthesis, **Figure 3**). By choosing these stages we aimed to capture the initiation, period of transition between cell division and cell expansion, then elongation of the spur. To ensure that the stages between the two species were comparable the stages were defined relative to anthesis (**Table 1**). In total there were 48 samples sequenced: two species sampled at three developmental stages, separated into dorsal and ventral petals, with four biological replicates each, where each biological replicate is an individual plant. Total RNA was extracted, and 10-15 million 150 bp paired-end reads were generated for each sample using the Novaseq 6000 platform. For each species, a *de novo* transcriptome assembly was generated using Trinity (see methods). To assess the quality of the assembly, reads were mapped back to each assembly using Bowtie2. At least 96% reads were mapped back to their respective assemblies. To check the fragmentation of the data, the ExN50 statistic was calculated for each species (**Figure S5**) and the scores are comparable to those from other published *de novo* transcriptomes (Carruthers et al., 2018). We then checked how the *de novo* assemblies represent known full-length protein-coding genes. There are 51,737 proteins in the *A. majus* reference proteome of which 44.0% (22,763 proteins) are represented by nearly full-length transcripts (having over 80% alignment coverage) from our *A. majus* assembly. Given that only petal tissue was sequenced, this indicates good sequencing coverage. In our *L. vulgaris* assembly there were 17,664 proteins that had over 80% alignment coverage. This lower figure was expected as the *L. vulgaris* sequences were being aligned to the *A. majus* proteome.

Differentially expressed genes between ventral and dorsal tissues were called by the DESeq2 R package. To check the quality of the micro-dissection, the expression patterns of dorsal-specific symmetry genes such as *DICHOTOMA* (*DICH*) and *CYCLOIDEA* (*CYC*) were examined (Luo et al., 1996; Luo et al., 1999). Expression was strictly confined to the dorsal tissue which suggests precise tissue dissection (**Figure S8**). To investigate the relationships between the samples, principal component analysis (PCA) was performed on all samples for each of the species (**Figures S6**; **S9**). In PC1, samples cluster by developmental stage in both species. In *A. majus* each developmental stage separates evenly, whilst in *L. vulgaris* the early and intermediate stages cluster closer together than the late stages. In both datasets, the ventral petals from each biological replicate cluster together, and the dorsal petals from each biological replicate cluster together, aside from one late-stage *A. majus* replicate. This is likely due to an error whilst processing the samples, and this sample was therefore removed from all further analysis. It was observed that the relative amount of variance between the ventral and dorsal petals is smaller than the variation between stages. In *L. vulgaris*, PC5 and PC6 separate the ventral and dorsal petals and comprise 4% of the total variance. Similarly, PC3 and PC4 separate the dorsal and ventral petals in *A. majus*, and account for 6% of the total variance. Finally, there is more variance between replicates in *L. vulgaris* than in *A. majus*. This is likely due to the fact that a highly inbred laboratory line was used for *A. majus*, whereas seeds from a wild population were used for *L. vulgaris*.

The number of statistically significantly differentially expressed genes (DE genes) between the ventral and dorsal petals across developmental stages gives insight into the dynamics of gene expression. In *A. majus*, there were 380 and 322 genes that were DE only at the early or intermediate time points respectively; there were 345 DE genes in both of these time points (**Figure S10**). In contrast, there were only 231 DE genes expressed only at the late stage. In *L. vulgaris*, there were 126 and 365 significantly DE genes at only the early and intermediate time points respectively; 251 significantly DE genes were shared between the two time points. There are more shared DE genes between the early and intermediate stages (251) than between the early and late stages (93) or intermediate and late stages (181). This is consistent with the PCA performed in *L. vulgaris* which indicates that the early and intermediate stages in *L. vulgaris* are more similar than the intermediate vs. late developmental stages (**Figure S9**). There were more DE genes in *A. majus* than in *L. vulgaris*. This may be because the variance of gene expression between biological replicates within one tissue is higher in *L. vulgaris* due to larger differences in genotypes between biological replicates than that in *A. majus*, and therefore requires larger difference in expression levels to reach statistical significance.

### 3.4 Examining global gene expression dynamics and spur-specific genes

To compare the expression patterns between ventral and dorsal tissues over time, genes were clustered by expression pattern (see methods). Genes were grouped into seven clusters with supervised clustering in both *L. vulgaris* and *A. majus* (**Figure S11**, **Tables S1**, **S2**). This revealed that gene expression patterns in *L. vulgaris* and *A. majus* are generally monotonic over time (within one tissue (the ventral or dorsal petal)). For example, when the expression level of a gene increased from early to intermediate stage, it rarely decreased from intermediate to late stage (**Figures S11**, **S12**). Each cluster contained a unique expression pattern; for example, cluster 2 shows consistently higher expression in the ventral petal than the dorsal petal and expression decreases with time. Conversely, cluster 5 contains genes where expression is consistently higher in the dorsal petal than the ventral petal and expression decreases with time. Such monotonicity suggests that the expression dynamics of the majority of genes are consistent in direction across the stages and the ventral-specific genes and dorsal-specific genes stay specific to their respective tissues across the developmental stages.

### 3.5 Spur genes linked to cell division are expressed early in spur development

**Figure 4:**
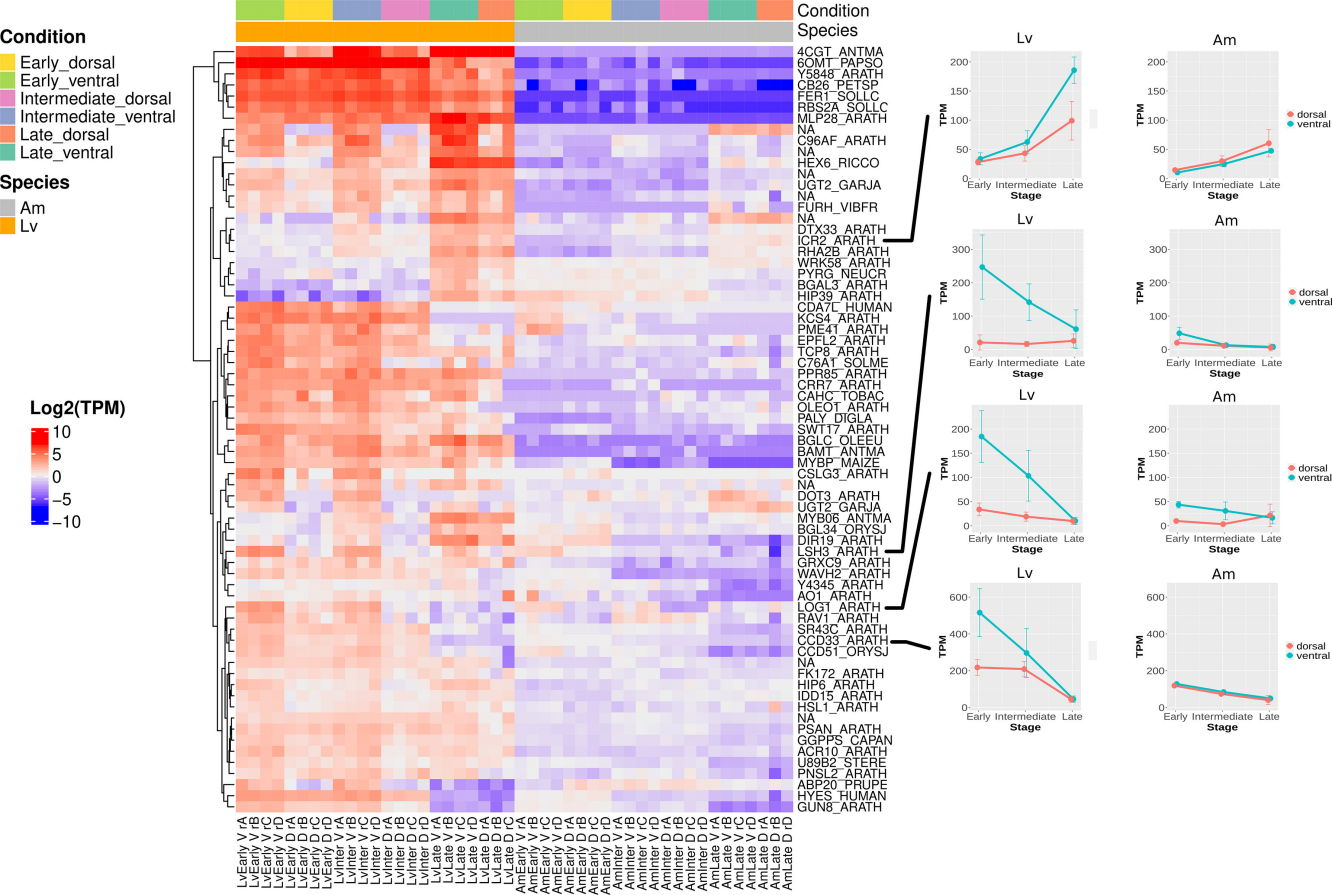
Heatmap showing the spur aid genes at all developmental stages in *L. vulgaris* and *A. majus*. Spur aid genes were clustered based on expression pattern. For each spur aid gene (see methods), raw read counts were transformed using transcripts per million (TPM) and then normalised by subtracting the median TPM across all samples (including both species) for each gene. Each row represents a gene, and each column a sample. Red indicates above the average, and blue indicates below average. Expression patterns of spur aid genes highlighted in text are shown on right hand side of heatmap.

To further classify genes that may be important for spur development in a systematic way, we took advantage of our experimental design and used the gibba of *A. majus* as a control to generate a list of spur-specific genes at the three developmental stages in *L. vulgaris*, as described in the methods. Including each developmental stage, this produced a list of 195 spur aid genes (**Figure 4**, **Table S3**) and 330 spur repression genes (**Figure S13**, **Table S4**). The number of spur aid genes at an early (61), intermediate (93) and late (99) stage was similar. However, there were fewer spur repressor genes at an early (94) stage than at intermediate (197) and late stages (134).

We hypothesised that spur aid genes which are expressed at an early stage in spur development are likely to be critical for establishing the patterning of the spur (**Figure 4**). A particularly interestingly spur gene is the *L. vulgaris* ortholog of *CYCLIN-D3-3* (*CCD33*) in *A. thaliana* (*LvCCD33*), which regulates cell division (Dewitte et al., 2007; Collins et al., 2012). Expression of this gene is high in the ventral petal compared to the dorsal petal at the early stage of spur development, and this trend persists at the intermediate stage, despite a smaller difference in expression levels. In the late stage, the difference decreases further and the expression levels become similar between ventral and dorsal petals. In contrast, the expression of the *A. majus* ortholog of *CCD33* (*AmCCD33*) is similar between the ventral and dorsal petals throughout gibba development. To investigate whether an increase in cyclin activity was broadly observed in *L. vulgaris* during spur development, activities of all potential cyclin genes were further investigated by checking the expression of all genes which had a cyclin N-terminal or C-terminal domain (Pfam database). Examination of the expression pattern of these genes in *L. vulgaris* reveals that there is also a ventral-petal specific expression pattern of *LvCCD33* and *LvCCD32* (**Figure S14**). This pattern is not observed in the other cyclins, suggesting that it is specifically *LvCCD32* and *LvCCD33* which exhibit differential expression between the ventral and dorsal petal. In *A. majus*, this pattern is observed at a much lower level (**Figure S15**).

Another spur gene which has a similar expression pattern to *LvCCD33* is the *A. thaliana* ortholog of *LONELY GUY 1* (*LOG1*) (**Figure 4**). The *LOG1* gene is one of nine genes in the LOG family in *A. thaliana*, and codes for an enzyme which is capable of directly converting cytokinin precursors into an active form (Kuroha et al., 2009; Tokunaga et al., 2012). Cytokinins are also linked to the control and induction of the cell cycle (Schaller et al., 2014). This gene is up-regulated in the ventral petal compared to the dorsal petal at an early and intermediate stage in *L. vulgaris*. When the ortholog of *LOG1* is examined in *A. majus*, an interesting expression pattern is revealed. Expression at an early and intermediate stage in the ventral petal is also higher than in the dorsal, however the expression levels in the ventral petal of *A. majus* are less than one third of those observed in *L. vulgaris.*

To gain insight into what may be regulating *LvCCD33* or *LvLOG1*, we examined the 13 spur aid genes that were suggested to be involved in the biological process of DNA-templated transcription (GO:0006351). One such spur aid gene is *FLORICAULA* (*FLO*), the *A. majus* ortholog of *LEAFY* (*LFY*) in *A. thaliana* (**Figure S16A**). *FLO* plays an important role in floral meristem development (Coen et al., 1990; Weigel et al., 1992; Moyroud et al., 2010). This gene shows a high level of expression in the ventral petal compared to the dorsal petal at an early and intermediate developmental stage in *L. vulgaris*. In contrast, the expression of *FLO* in *A. majus* was not detected. Intriguingly, the Zinc-finger transcription factor *POPOVICH* (*POP*), which was recently suggested to be necessary for spur development in *Aquilegia*, was also captured in our list of spur aid genes associated with transcription (**Figure S16B**) (Ballerini et al., 2020). The *TCP* transcription factor *TCP4* was not identified as a spur gene (*TCP4* was previously implicated in spur development in *Aquilegia* by Yant et al., 2015), however we did identify a member of the same gene family, the *A. thaliana* ortholog of *TCP8* as a spur aid gene (**Figure S16C**). Transcription factors associated with organogenesis are also present in our spur gene list. For example, the *A. thaliana* ortholog of *LIGHT-DEPENDENT SHORT HYPOCOTYLS 3/4* (*LSH3/4*) (**Figure 4**), gene shown to be involved in lateral organ outgrowth (Cho and Zambryski, 2011). Also present is the *A. thaliana* ortholog of Zinc finger protein *SHOOT GRAVITROPISM 5* (*IDD15*) (**Figure S16D**), which can affect organ orientation through auxin action (Cui et al., 2013).

### 3.6 Different cell wall remodelling processes are found in the spur at different development stages

At the early, intermediate and late stages in spur development, multiple spur genes are associated with cell wall remodelling. We can examine which expression cluster contains a particular spur aid gene (**Figure S11**). For example, we considered the spur aid genes *ENDOGLUCANASE 8* (*GUN8*) (**Figure S17A**) and *SUGARS WILL EVENTUALLY BE EXPORTED TRANSPORTERS 17 (SWT17)* (**Figure S17B**). *GUN8* is involved in cellulose deposition (Tsabary et al., 2003), and *SWT17* codes for a fructose transporter in *A. thaliana* (Guo et al., 2014). These genes are located in cluster 2, a cluster which contains genes which have consistently higher expression in the ventral petal than the dorsal petal across all stages, and expression levels decrease over time in both tissue types.

Spur aid genes associated with cell wall remodelling and maturation are also present in cluster 1. This cluster includes genes which also exhibit higher expression in the ventral than the dorsal petal, however the expression of genes increases as the spur matures (**Figure S11**), such as *HEXOSE CARRIER PROTEIN HEX6* (*HEX6*), which is also associated with the transport of sugar (**Figure S17C**) (Weig et al., 1994). Two spur genes associated with cell expansion and maturation found in cluster 1 include *INTERACTOR OF CONSTITUTIVE ACTIVE ROPS 2* (*ICR2*), a gene associated with cytoskeleton reorganisation (**Figure 4**) (Lavy et al., 2007) and Alkane hydroxylase MAH1 (*C96AF*) (**Figure S17D**), a gene observed in *A. thaliana* to be involved in the production of wax on the cuticle (Greer et al., 2007). Therefore, our spur gene and cluster analysis can be used to complement each other.

### 3.7 GO enrichment analysis reveals dynamic expression of plant hormones during spur development

**Figure 5:**
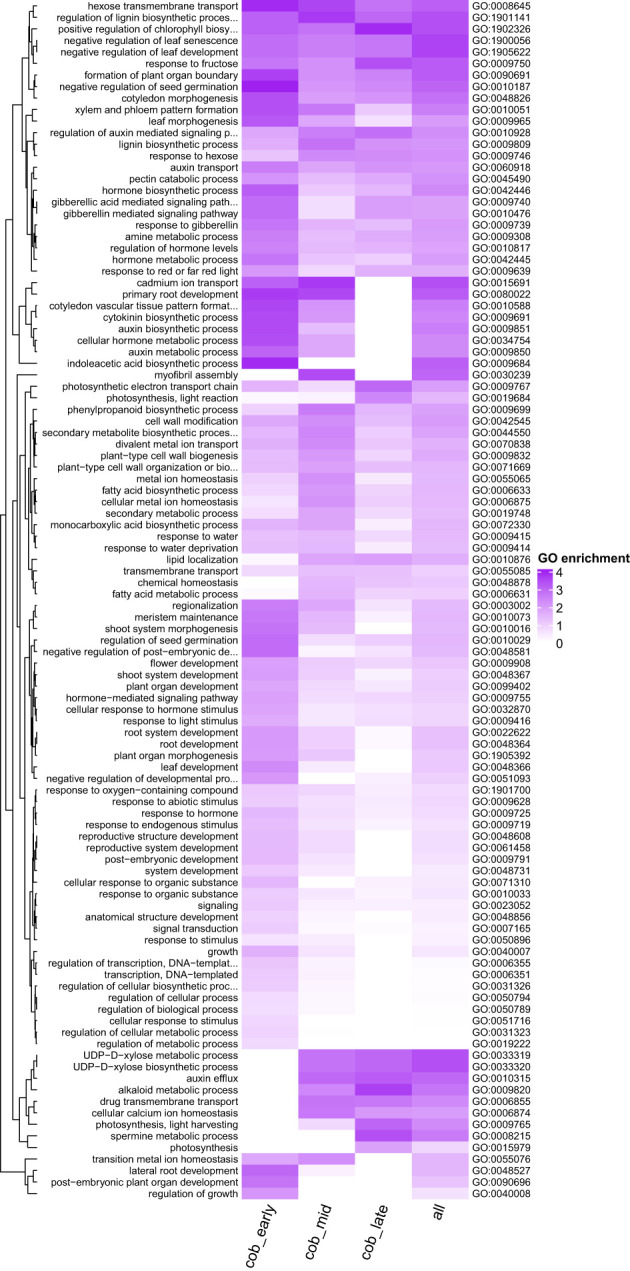
Heatmap shows GO terms for biological process that are enriched in spur genes (see methods) across different developmental stages. P value cutoff is < 0.005 and the node size is 5 for visualisation purposes. Each row represents a GO term, and each column a gene set. cob_early = both spur aid and suppressor genes at the early stage, cob_mid = both spur aid and suppressor genes at the intermediate stage, cob_late = both spur aid and suppressor genes at the late stage and all = spur aid and suppressor genes from all three developmental stages. Dark purple indicates a higher level of enrichment.

**Figure 6:**
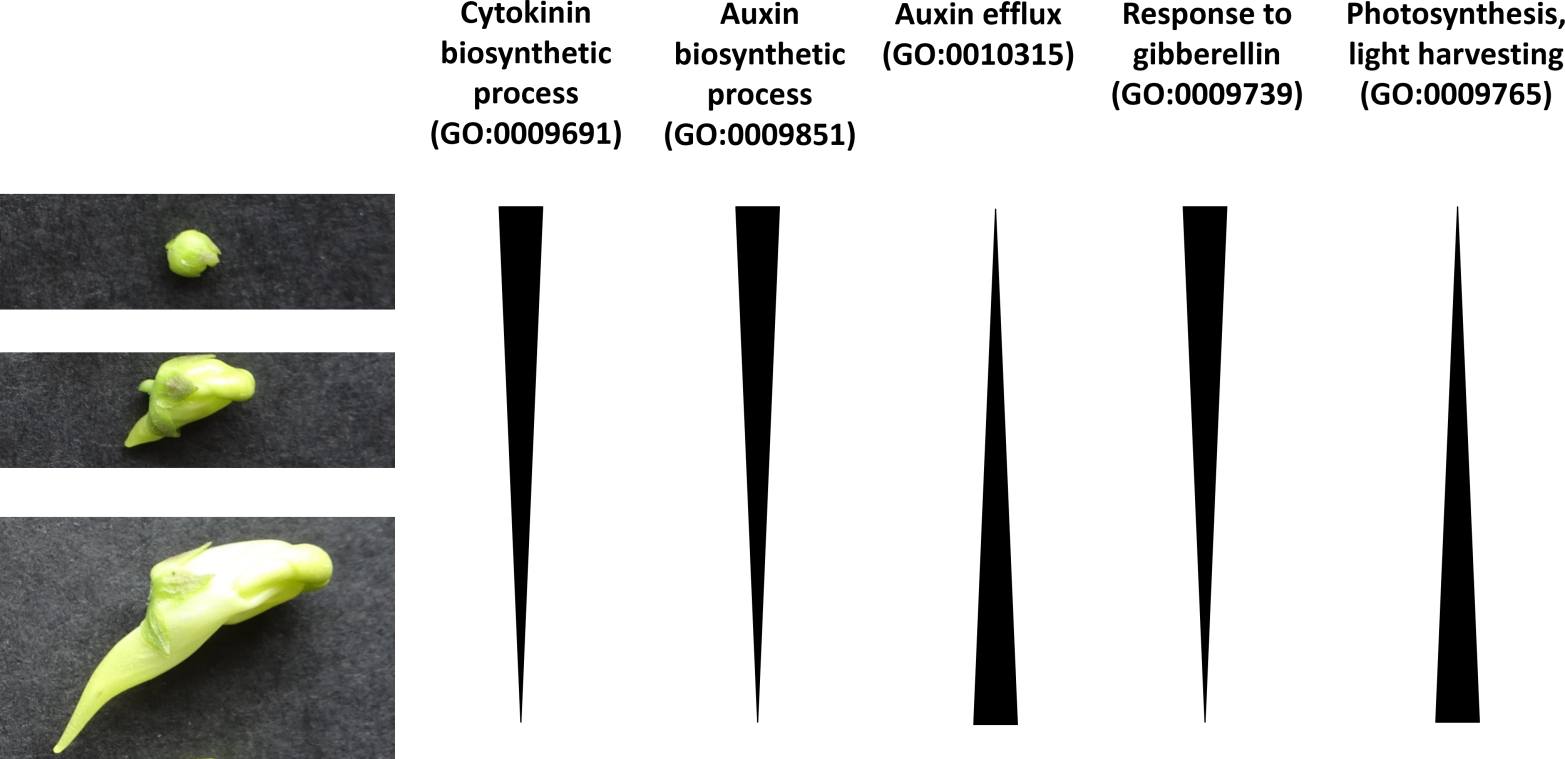
Cartoon which suggests patterns of GO enrichment across the three developmental stages in spur development of *L. vulgaris*. The three stages of spur development are shown on the left-hand side (early, intermediate and late from top to bottom).

In order to gain more insights into the biological pathways which may be important in spur development, a GO enrichment analysis was performed on the spur genes at each developmental stage, as well as combined (**Figure 5**). GO terms associated with photosynthesis are significantly enriched in spur genes (photosynthesis, light harvesting (GO:0009765)). Multiple plant hormone pathways were found to be enriched amongst the spur genes, however the expression patterns for different hormones differed. Cytokinin biosynthetic process (GO:0009691) was found to be significantly enriched at an early stage and a decrease in enrichment score was apparent throughout development. Auxin biosynthetic process (GO:0009851) and response to gibberellin (GO:0009739), followed this trend of a decrease in enrichment score through developmental time. On the other hand, genes associated with auxin efflux (GO:0010315) were not enriched in the early stage, but enriched in the later stages (**Figure 6** summarises these trends).

## 4 Discussion

### 4.1 RNAseq data reflect morphological observations

Microscopy data indicate that spur development in *L. vulgaris* at an early stage is characterised by a phase of cellular division, whereas cell expansion is more dominant at the late stage. In *A. majus* both cell division and cell expansion occur across the developmental stages. Given that the gibba is established earlier than the spur, it could be that the developmental program of the gibba is established earlier. When our RNAseq data (in which three time points were examined) was investigated using PCA, these trends were echoed – the early and intermediate *L. vulgaris* stages were more similar to each other than to the late stage, whereas the *A. majus* tissues were consistently spaced. There were also more differentially expressed genes between the ventral and dorsal tissues, shared between the early and intermediate *L. vulgaris* stages than with the later stages. Combined, our morphological and molecular observations suggest that the growth profile of the *L. vulgari*s spur is dynamic, whereas in the *A. majus* gibba it is slower and steadier. In *Aquilegia coerula*, an initial period of cell division was also observed in the developing spur, which transitioned to cell expansion when spurs were approximately 1 mm (Yant et al., 2015). Both *Linaria* and *Aquilegia* therefore exhibit a phase of cell division and a phase of cell expansion during spur development. It may be that the duration or rate of the two phases differ between the two systems.

### 4.2 Spur-specific genes associated with cell proliferation

The discovery of a ventral-specific expression pattern of genes associated with cell proliferation (*LvCCD33* and *LvLOG1*) early in spur development is intriguing and correlates with our morphological observations that an increase in cell number occurs early in spur development. In *A. thaliana* cytokinins have been shown to increase the expression of and activate D-type cyclins, which in turn promote cell division (Riou-Khamlichi et al., 1999; Dewitte et al., 2007). In *A. majus*, two out of three of the D-type cyclins studied also exhibited an increase in expression in response to cytokinin application (Gaudin et al., 2000). Further, given that a boundary is required in the development of a lateral organ (to restrict cell proliferation to the nascent spur), locally reduced cell proliferation may be required. Perhaps the expression of the transcription factor *CYCLOIDEA* (*CYC*) gene or its downstream targets in the dorsal tissue restrains expression of *LvCCD33* to the ventral petal and developing spur (Luo et al., 1996). *LSH3* is also known as *ORGAN BOUNDARY1* (*OBO1*). In *A. thaliana*, *OBO1* has been shown to be important for the generation of boundaries in floral organ development (Cho and Zambryski, 2011). It is of interest that in *A. thaliana LSH3* is activated by *CUP-SHAPED COTYLEDON1* (*CUC1*), a gene that is known to be important to generate boundaries in the *A. thaliana* flower (Aida et al., 1997; Takeda et al., 2011). Finally, the identification of the *A. thaliana* ortholog of *IDD15* (encoding a transcription factor associated with gravitropism) is also intriguing, as the developing spur requires direction (Cui et al., 2013).

Transcription factors associated with indeterminacy were also identified. *FLO* is a gene that is essential for the formation of the floral meristem in both *A. majus* and *A. thaliana* (Coen et al., 1990; Weigel et al., 1992). It was identified as a ‘spur aid’ gene and yet demonstrates a striking lack of expression in *A. majus*. It is interesting to consider that *FLO* may have been co-opted in *L. vulgaris* to have a role in spur outgrowth. However, a similar recruitment event has not been identified yet for *FLO* in any other species within the plant kingdom. The ortholog of *POP*, which was recently shown to increase the number of cells in the *Aquilegia* spur, was also identified as a spur aid gene in our study (Ballerini et al., 2020). It is interesting that this gene may be involved in spur development in both *L. vulgaris* and *Aquilegia*. Previous studies have suggested that the transcription factor *TEOSINTE BRANCHED 4*/*CYCLOIDEA*/*PCF* (*TCP4*) plays an important role in nectar spur development in *A. coerulea* (Yant et al., 2015), and that the *KNOX* genes *HIRZINA* (*HIRZ*) and *INVAGINATA* (*INA*) are linked to spur development in *L. vulgaris* (Golz et al., 2002; Box et al., 2011). The above genes were not identified as differentially expressed in our spur-specific gene set. We speculate that the early stage in our analysis was perhaps not early enough to capture the expression of these genes. We did identify a related *TCP* transcription factor, the *A. thaliana* ortholog of *TCP8*, as a spur aid gene, which may promote endoreduplication in the leaf of *A. thaliana* (Aguilar-Martínez and Sinha, 2013; Zhang et al., 2019). Given the high expression of this gene at an early stage, we hypothesise that it could be involved in cell division in the *L. vulgaris* spur.

### 4.3 Spur-specific genes associated with cell wall remodelling

The outgrowth of a lateral organ such as the spur requires active cell wall remodelling (Monniaux and Hay, 2016; Anderson and Kieber, 2020). Cellulose deposition occurs in the primary cell wall, and pre-patterns the later accumulation of lignin and other components of the secondary cell wall. Early in spur development, the expression of the spur aid gene *LvGUN8*, which is linked to the placement of cellulose, was observed (Tsabary et al., 2003). At a later stage, the spur expands. Cell expansion involves the transport of sugars into the vacuole, which encourages more water to move into the vacuole through osmosis and allows the cell to expand. The spur aid genes *LvSWT17* and *LvHEX6* encode sugar transporters (Weig et al., 1994; Guo et al., 2014), expressed at an early and late stage respectively. Their expression may prepare and enable the spur to rapidly expand by changing the water potential inside cells. The observation that expression of *LvICR2* (involved in cytoskeleton reorganization in *A. thaliana*) occurs when our morphological data suggest that the spur is rapidly expanding may indicate that this gene plays an important role in cytoskeleton reorganization in spur development (Lavy et al., 2007). As the spur matures, the cuticle matures and waxes may be produced. Indeed, the spur aid gene *LvC96AF* is highly expressed at a late stage, a gene observed in *A. thaliana* to be involved in the production of wax on the cuticle (Greer et al., 2007). Overall, our results imply that different cell wall modification changes may be required at different stages of spur development.

### 4.4 Our GO analysis suggests that plant hormones may play a role in spur development in Linaria

Plant hormones and interactions between plant hormones can control the development of indeterminate areas of growth in *A. thaliana*, such as the shoot and root apical meristem (Vanstraelen and Benkov, 2012). For example, in the shoot and floral meristem, auxin accumulates at the site of developing organs (Heisler et al., 2005; Xu et al., 2018) and the termination of the floral meristem is enabled through the restriction of cytokinin via auxin (Zhang et al., 2018). The above plant hormones have also been shown to play a role in the positioning of organs, such as the lateral roots of *A. thaliana* (de Smet et al., 2010; Chang et al., 2015). Given that biosynthesis of auxin and cytokinin exhibit a higher enrichment score at an early stage in spur development in *L. vulgaris*, perhaps they are also playing a role to ensure a successful balance between proliferation and differentiation, and the proper positioning of the spur on the flower. Gibberellin (GA) has been linked to cell expansion in the roots and hypocotyls of *A. thaliana* (Rizza et al., 2017). The GO term response to GA also shows a higher enrichment score at an earlier stage; perhaps GA could play a role in the transition from cell division to cell expansion in the spur.

The GO terms for auxin efflux and photosynthesis, light harvesting have a higher enrichment score at a later stage in spur development. Auxin has been demonstrated to be involved in nectar spur elongation (through anisotropic growth) in *Aquilegia* (Zhang et al., 2020). Perhaps auxin efflux is also involved in spur elongation in *L. vulgaris*. The GO term photosynthesis, light harvesting was also found to be enriched in *Aquilegia* in a core set of ‘spur-specific’ genes (Ballerini et al., 2019). They reasoned that this may be because they compared the petal of a non-spurred species which was covered by the sepals, to the petal of a spurred species in which the spur was unprotected from the light. The dorsal petal of *L. vulgaris* is also partially covered by the calyx, whilst the ventral petal and spur is unprotected from the light. Therefore, the enrichment we observe may simply be due to the nature of our comparison, rather than revealing the biological involvement of photosynthesis-related genes in nectar spur development. Overall, our results show the enrichment of different plant hormones during different stages of spur development, which enables hypotheses to be generated and tested in the future.

## 5 Conclusions

Our combined morphological and transcriptomic datasets indicate that cell division is an important factor in nectar spur development in *Linaria*, and cell expansion is important later in spur development. Two different bioinformatic approaches were used (supervised clustering and a subtraction analysis) to identify key differentially expressed genes in spur development in *L. vulgaris* for the first time. These genes are good candidates for spur outgrowth in this species and can be further investigated in future studies, either through functional or genetic approaches.

## Data availability statement

The names of the repository/repositories and accession number(s) can be found below: https://www.ebi.ac.uk/ena PRJEB52140, https://doi.org/10.17863/CAM.90531 Raw gene counts, https://gitlab.com/g5337/spur_transcriptome, Code.

## Author contributions

BJG conceived the research plan. EC performed experiments. EC and QW analysed the data. EC wrote the first draft of the manuscript. QW and BJG edited the manuscript. All authors contributed to the article and approved the submitted version.
