# Supplementary material for: How do you build a nectar spur? A transcriptomic comparison of nectar spur development in Linaria vulgaris and gibba development in Antirrhinum majus
Source: Front Plant Sci. 2023 Jun 23;14:1190373. doi: 10.3389/fpls.2023.1190373 (PMC10328749; doi:10.3389/fpls.2023.1190373)
Supplement: Supplementary file 5 [file DataSheet_5.docx]

Supplementary Material

How do you build a nectar spur? A transcriptomic comparison of nectar spur development in *Linaria vulgaris* and gibba development in *Antirrhinum majus*

Erin Cullen^*^, Qi Wang, Beverley Glover^*^

*** Correspondence:** Erin Cullen, Beverley Glover: ecullen@mpipz.mpg.de, bjg26@cam.ac.uk

# Supplementary Figures and Tables

## Supplementary Figures


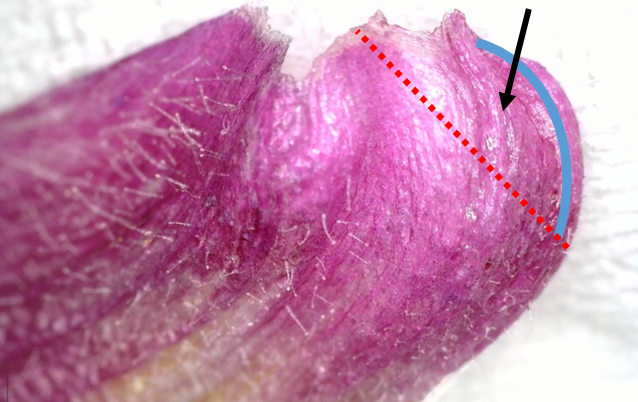


**Supplementary Figure 1.** How the gibba was defined in *A. majus*. Black arrow points to gibba. Red line indicates where the gibba was defined to end. Blue line indicates where the cells would be counted as within the gibba. To account for instances where an uneven edge was generated by the removal of the corolla, cells were counted from where the curvature of the gibba began to where it ended. A line was consistently drawn along the perimeter of the curve. This criterion was used at each developmental stage.


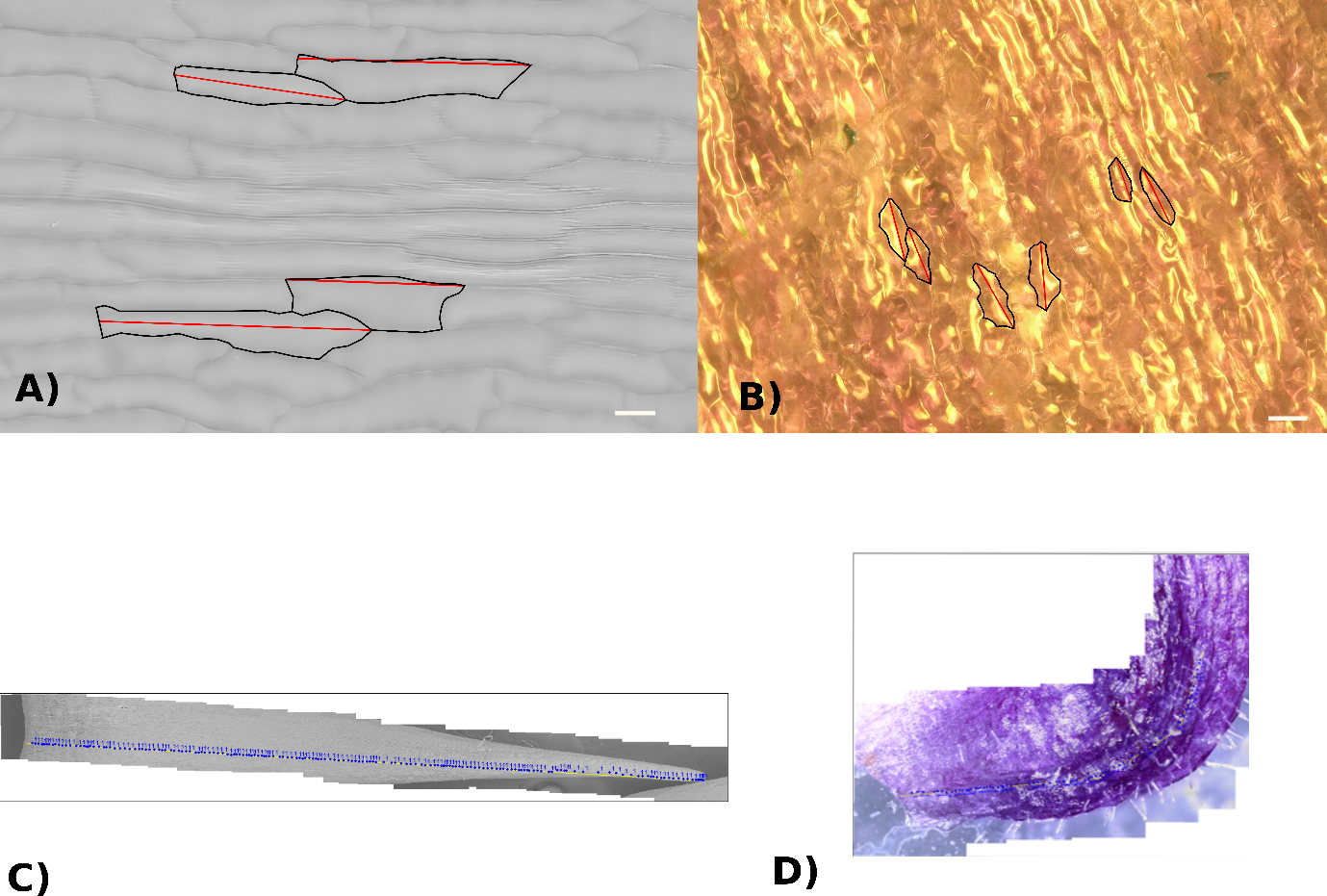


**Supplementary Figure 2.** Examples of how cell length and number were measured in the spur or gibba. (A, B) Cell outlines in black, red line indicates where cell length measurement taken. (A) Cryo-SEM image at the base of mature *L. vulgaris* spur. Scale bar represents 20 μm. (B) Keyence image of mature *A.majus* gibba. Scale bar represents 50 μm. (C) Merged cryo-SEM image of the mature *L. vulgaris* spur. (D) Merged Keyence image of mature *A.majus* gibba.


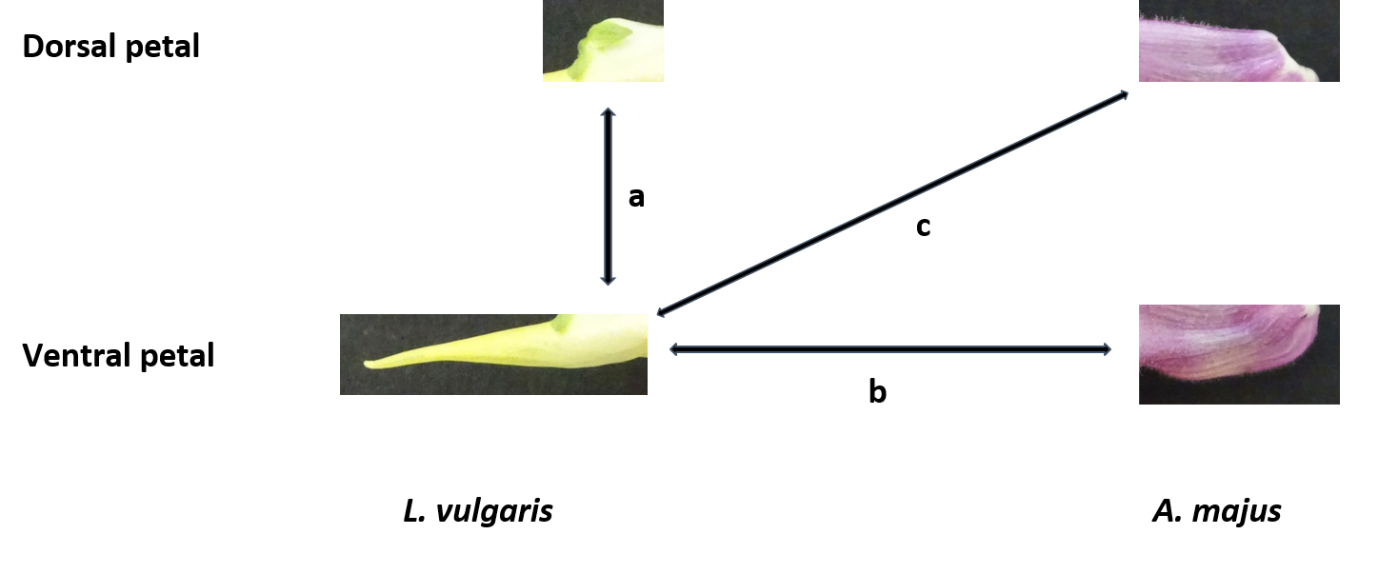


**Supplementary Figure 3.** Experimental design of the spur gene analysis. The corollas of both *L. vulgaris* and *A. majus* were dissected into the dorsal and ventral halves, and the lobes of the petals were dissected to remove any folding factors. A gene is defined as a ‘spur gene’ if it meets all of the following three criteria in at least one of the three developmental stages: a. Differentially expressed between ventral and dorsal tissues in *L. vulgaris* in this stage; b. Differentially expressed in ventral tissues comparing *L. vulgaris* and its *A. majus* ortholog in this stage; c. Among the relevant expressions in the three tissues of this stage, i.e., *L. vulgaris* ventral, *L. vulgaris* dorsal, and *A. majus* ventral, if the expression in *L. vulgaris* ventral is the highest among the three and the difference of mean log2-based TPM in *L. vulgaris* ventral vs. that in *A. majus* dorsal is greater than the cutoff of 1.0 it is defined as a spur aid gene of that stage; if *L. vulgaris* ventral is the lowest among the three and the difference of mean log2-based TPM in *L. vulgaris* ventral vs. that in *A. majus* dorsal is less than the cutoff of -1.0, the gene is defined as a spur suppress gene of that stage. Spur genes are the combination of spur aid genes and spur suppress genes.


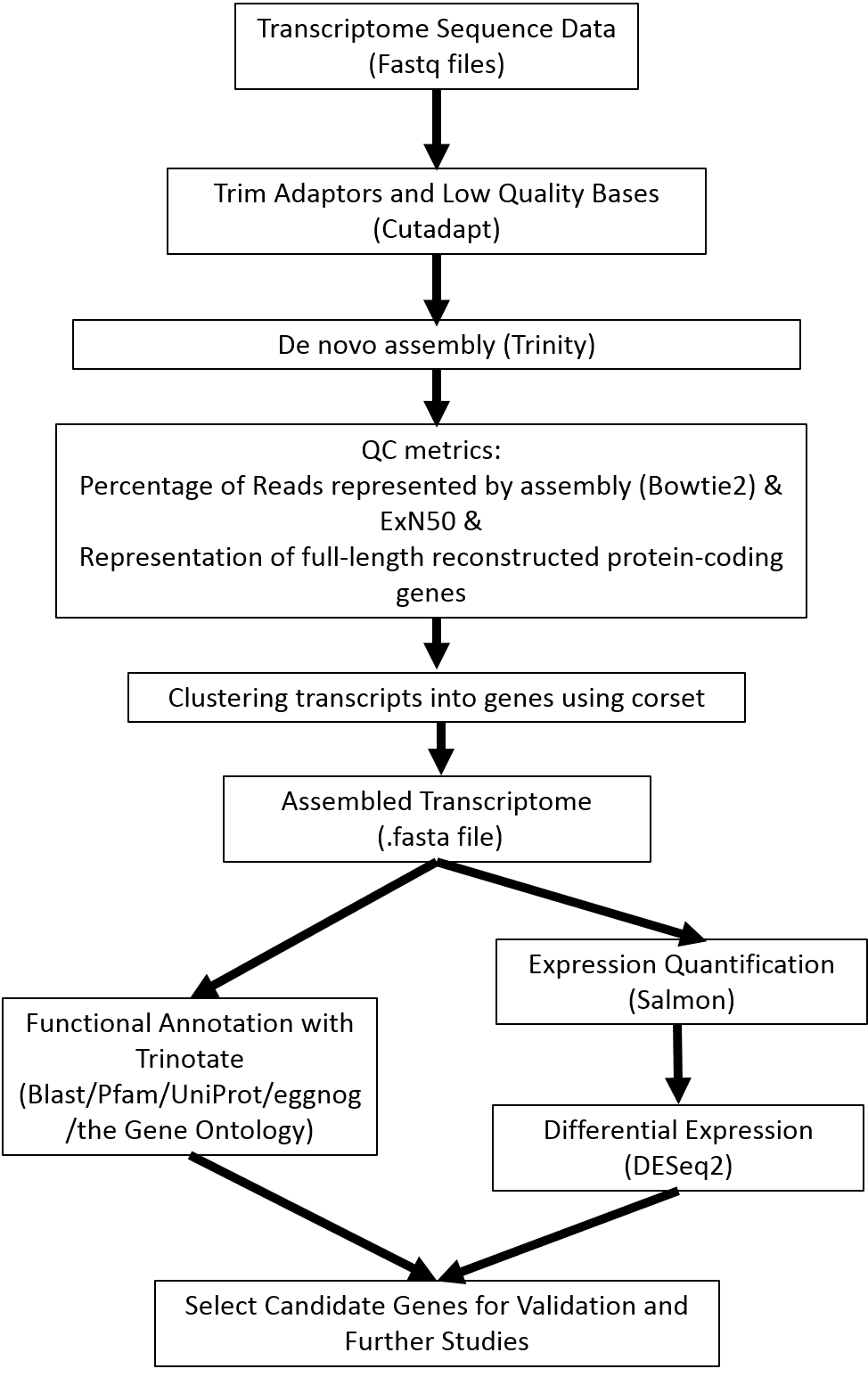


**Supplementary Figure 4.** Transcriptome analysis workflow.


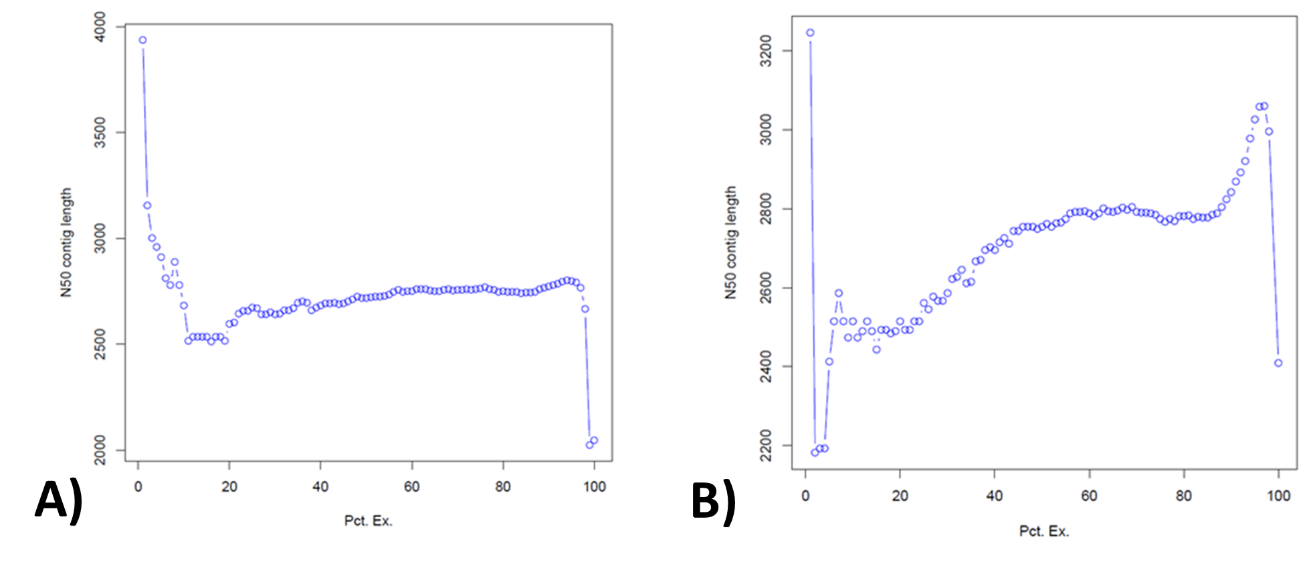


**Supplementary Figure 5.** ExN50 plots for the full assemblies of (A) *L. vulgaris* (statistic 2721). (B) *A. majus* (statistic 2755).


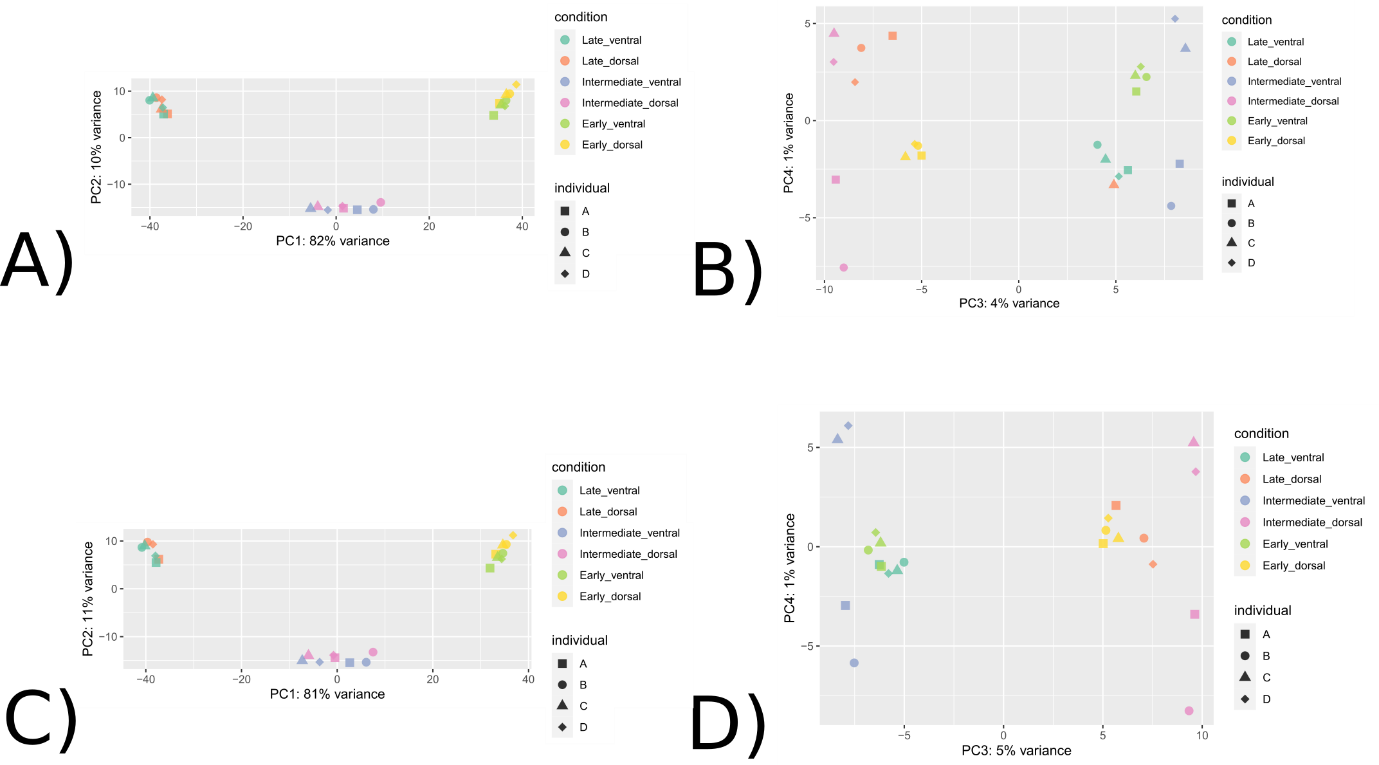


**Supplementary Figure 6.** Principal components analysis of whole transcriptome dataset from *A. majus*. Data transformed with variance stabilising function in DESeq2 prior to PCA. (A) Principal components one and two with all four biological replicates. (B) Principal components three and four with all four biological replicates. (C) Principal components one and two with outlier removed. (D) Principal components three and four with outlier removed.


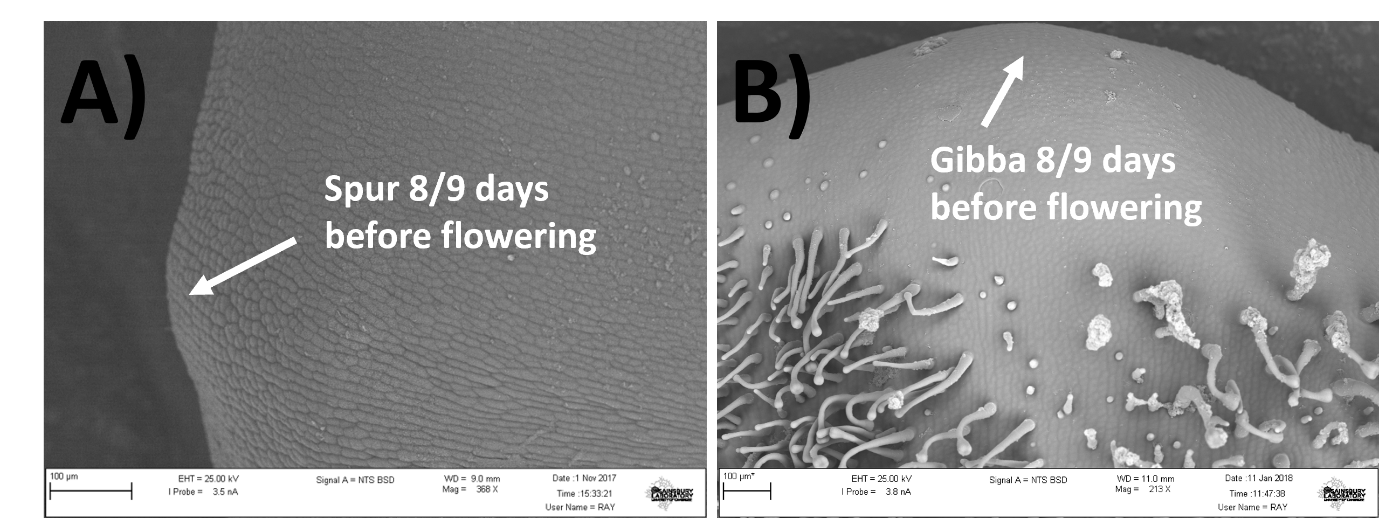


**Supplementary Figure 7.** Cryo-SEM images. (A) *L. vulgaris* spur at the stage eight or nine days prior to flowering. (B) *A. majus* gibba at the stage eight or nine days prior to flowering.


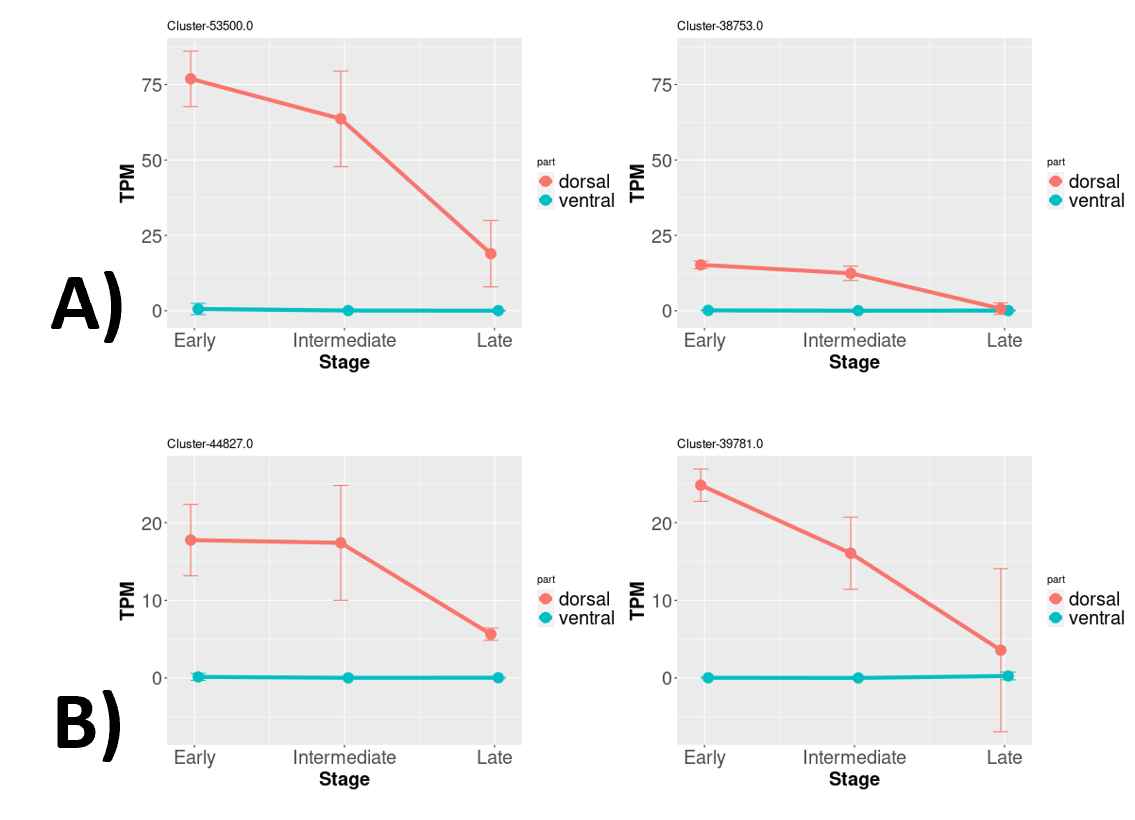


**Supplementary Figure 8.** Figure showing expression of the dorsal-specific genes *CYCLOIDEA* (*CYC*) and *DICHOTOMA* (*DICH*) at each developmental stage. On the left hand side expression profiles of *L. vulgaris* are shown, on the right hand side expression of the ortholog in *A. majus* is shown. Bars indicate 95% confidence intervals. Annotation is from the top BlastX hit to Uniprot. (A) *CYC.* (B) *DICH*.


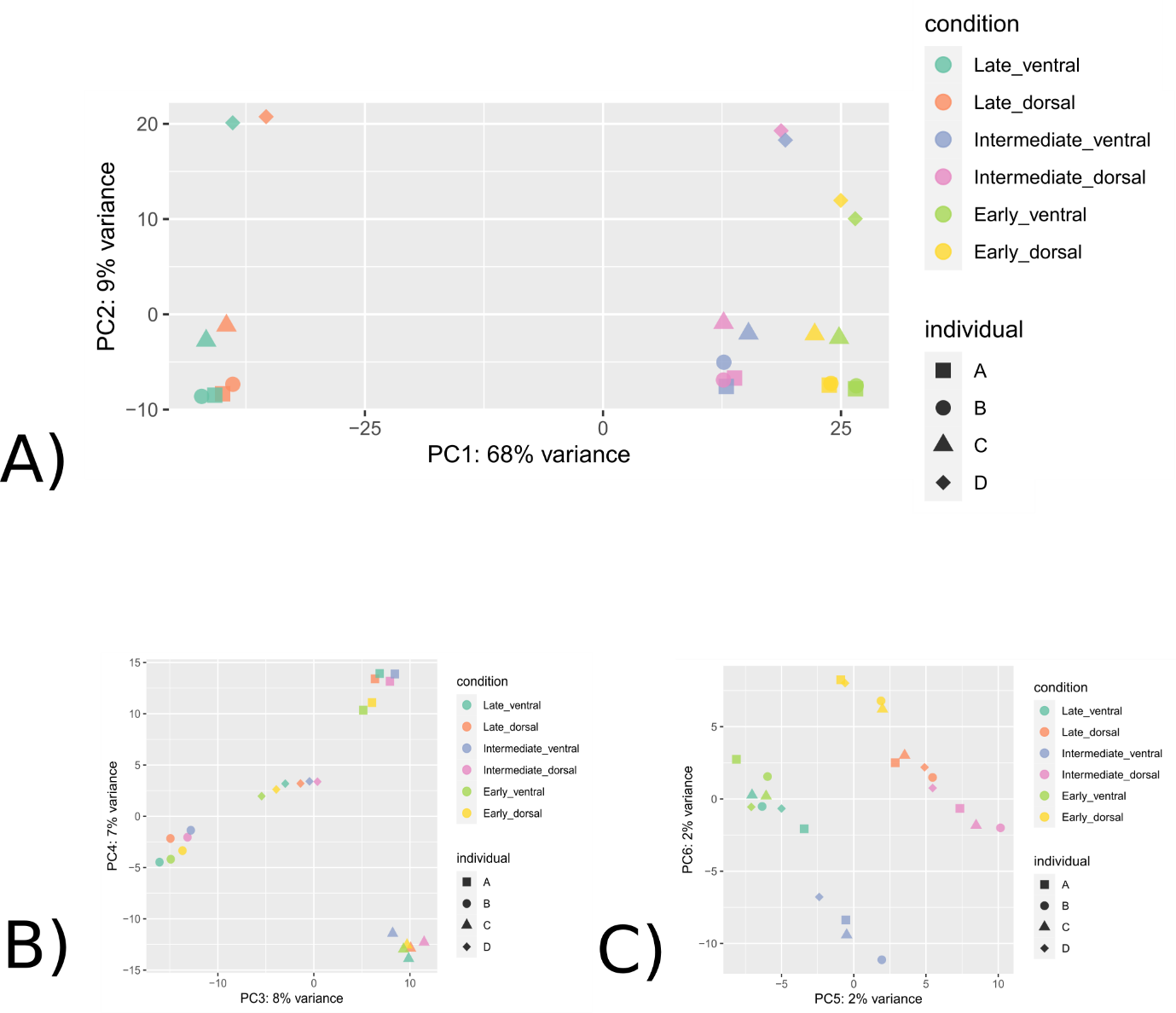


**Supplementary Figure 9.** Principal components analysis of whole transcriptome dataset from *L. vulgaris*. Data transformed with variance stabilising function in DESeq2 prior to PCA. (A) Principal components one and two. (B) Principal components three and four. (C) Principal components five and six.


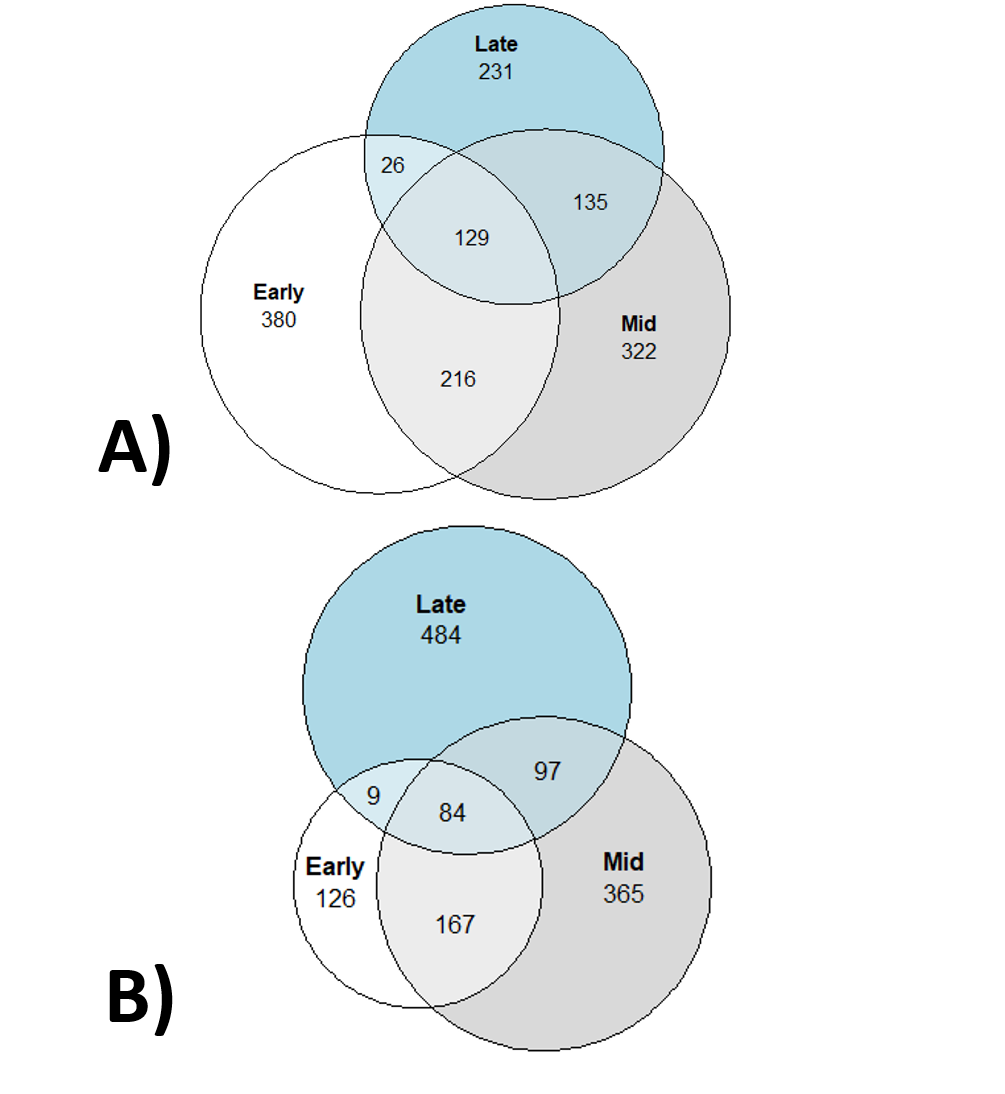


**Supplementary Figure 10.** Venn diagrams showing the number of differentially expressed genes between the ventral and dorsal petal in *L. vulgaris* and *A. majus*. Genes showing the absolute log2 fold change (l2fc) >1 and adjusted p-value <0.1 are shown. White = early in development; grey = intermediate in development; blue = late in development. (A) *A. majus*. (B) *L. vulgaris*.


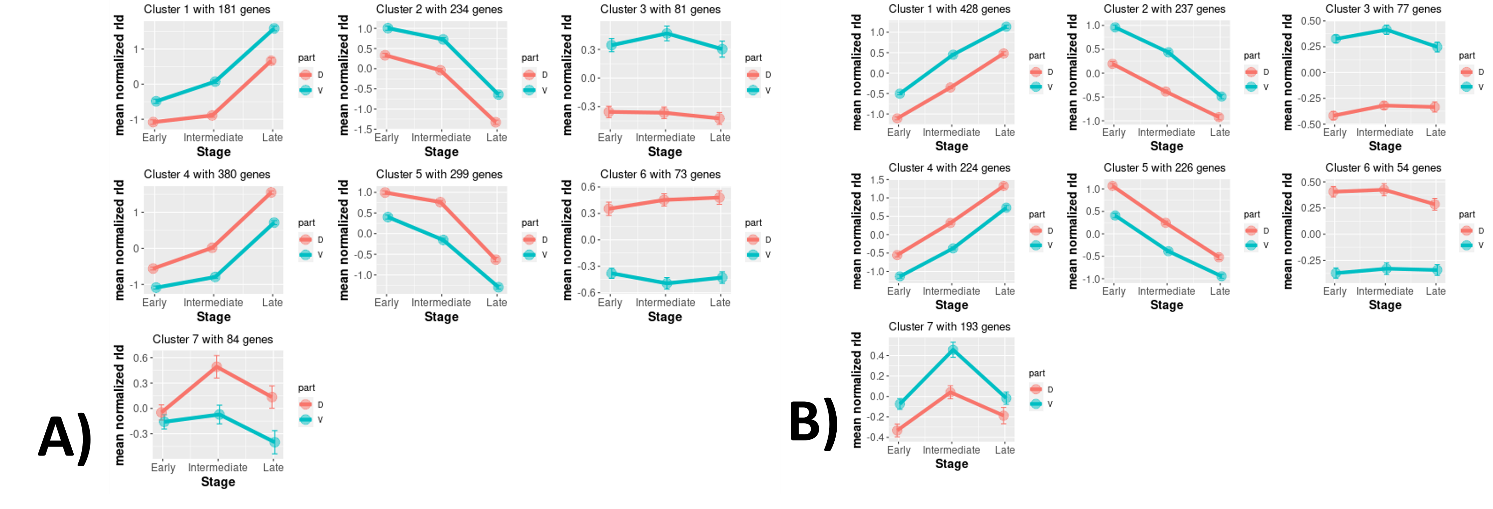


**Supplementary Figure 11.** Average expression patterns within each of the seven clusters of (A) *L. vulgaris* and (B) *A. majus*. Read counts were transformed with the regularised log transformation (rld) function in DESeq2 and then normalised by subtracting the mean rld across all samples for each gene. Each filled circle displays the mean of the normalised rld of all genes including all biological replicates in the corresponding cluster at each developmental stages of the tissue and the error bar shows the 95% confidence interval assuming normal distribution.


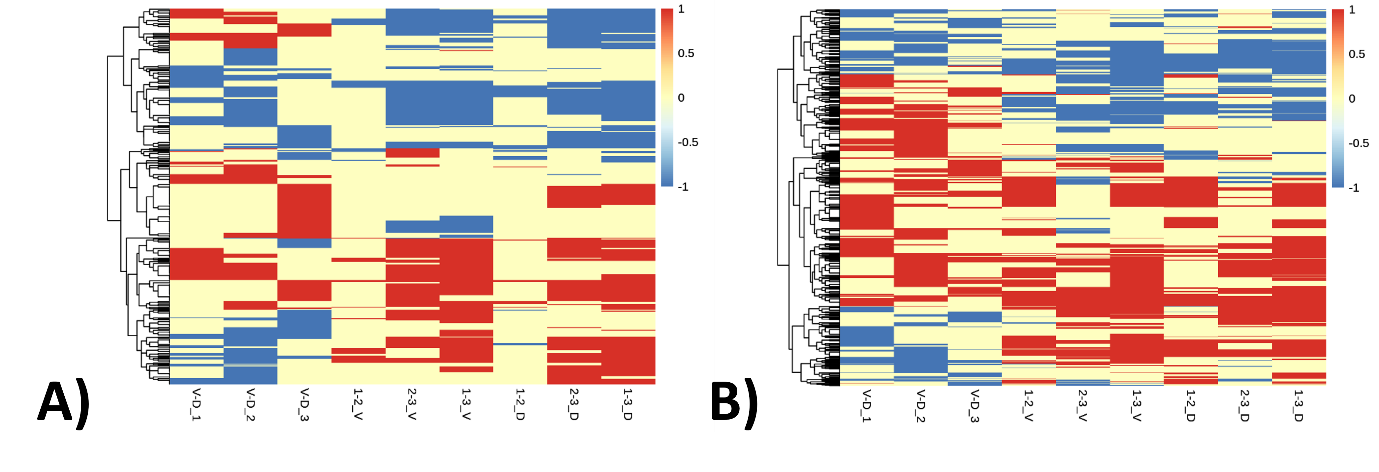


**Supplementary Figure 12.** Heatmap of expression patterns in (A) *L. vulgaris* and (B) *A. majus*. Expression patterns for the binned heatmap in *L. vulgaris*. V-D = ventral vs dorsal; _V = ventral vs ventral; _D = dorsal vs dorsal; 1 = earliest stage; 2 = intermediate stage; 3 = late stage. Red = highly DE, blue= lowly DE. Each row is a gene and each column is a comparison.


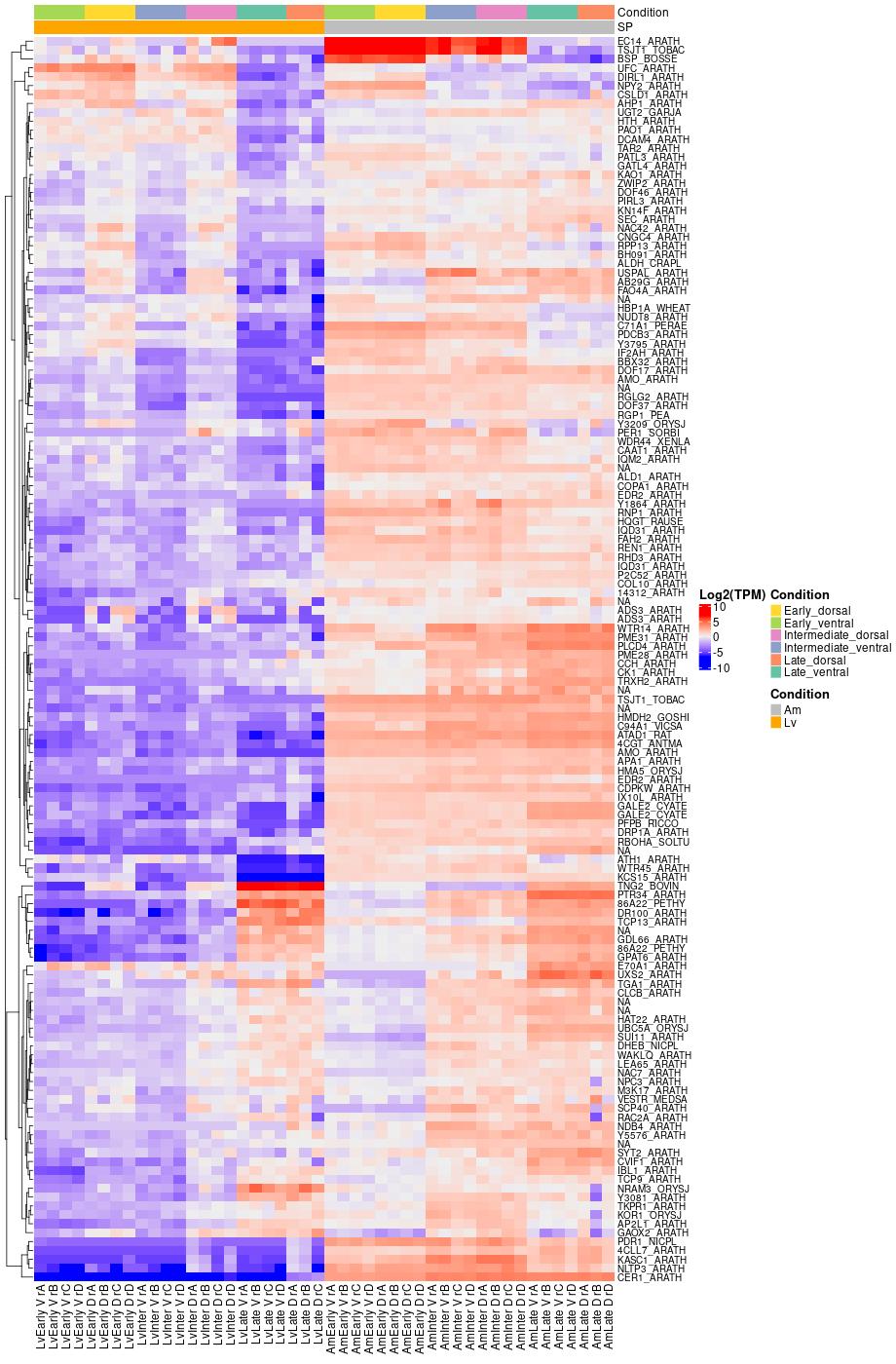


**Supplementary Figure 13.** Heatmap showing the spur suppressor genes at all developmental stages in *L. vulgaris* and *A. majus*. Spur suppressor genes were clustered based on expression pattern. For each spur suppressor gene (see methods), raw read counts were transformed using transcripts per million (TPM) and then normalised by subtracting the median TPM across all samples (including both species) for each gene. Each row represents a gene, and each column a sample. Red indicates above the average, and blue indicates below average.


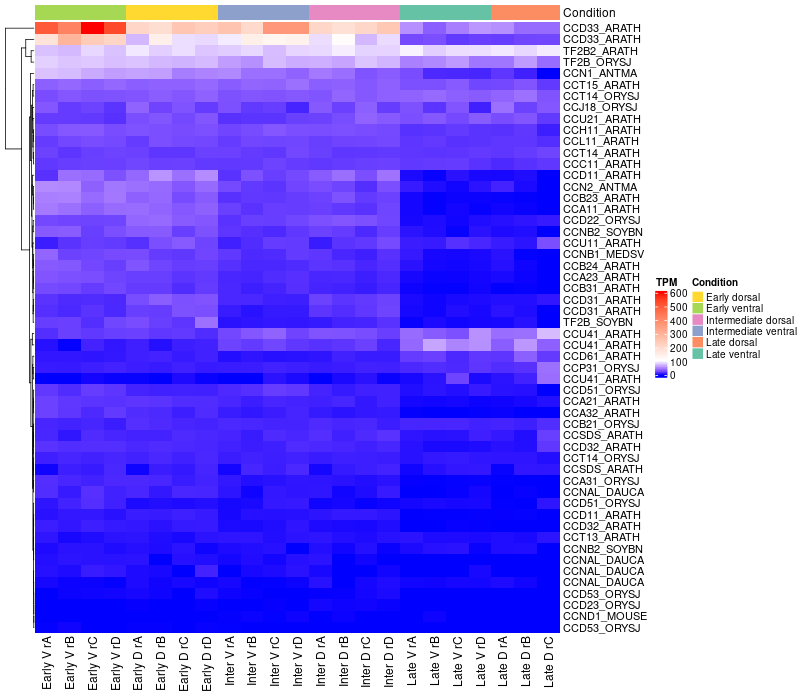


**Supplementary Figure 14.** Heatmap of all genes in *L. vulgaris* which have either an N-terminal or C-terminal cyclin domain, or both. Expression is plotted in transcripts per million (TPM). The annotation provided on the right hand side is the top BlastX hit to Uniprot.


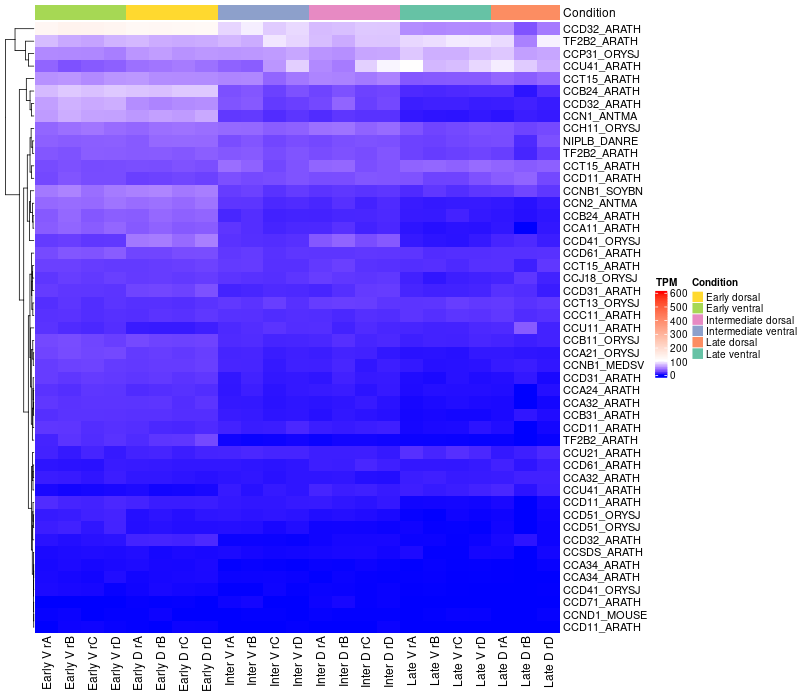


**Supplementary Figure 15.** Heatmap of all genes in *A. majus* which have either an N-terminal or C-terminal cyclin domain, or both. Expression is plotted in transcripts per million (TPM). The annotation provided on the right hand side is the top BlastX hit to Uniprot.


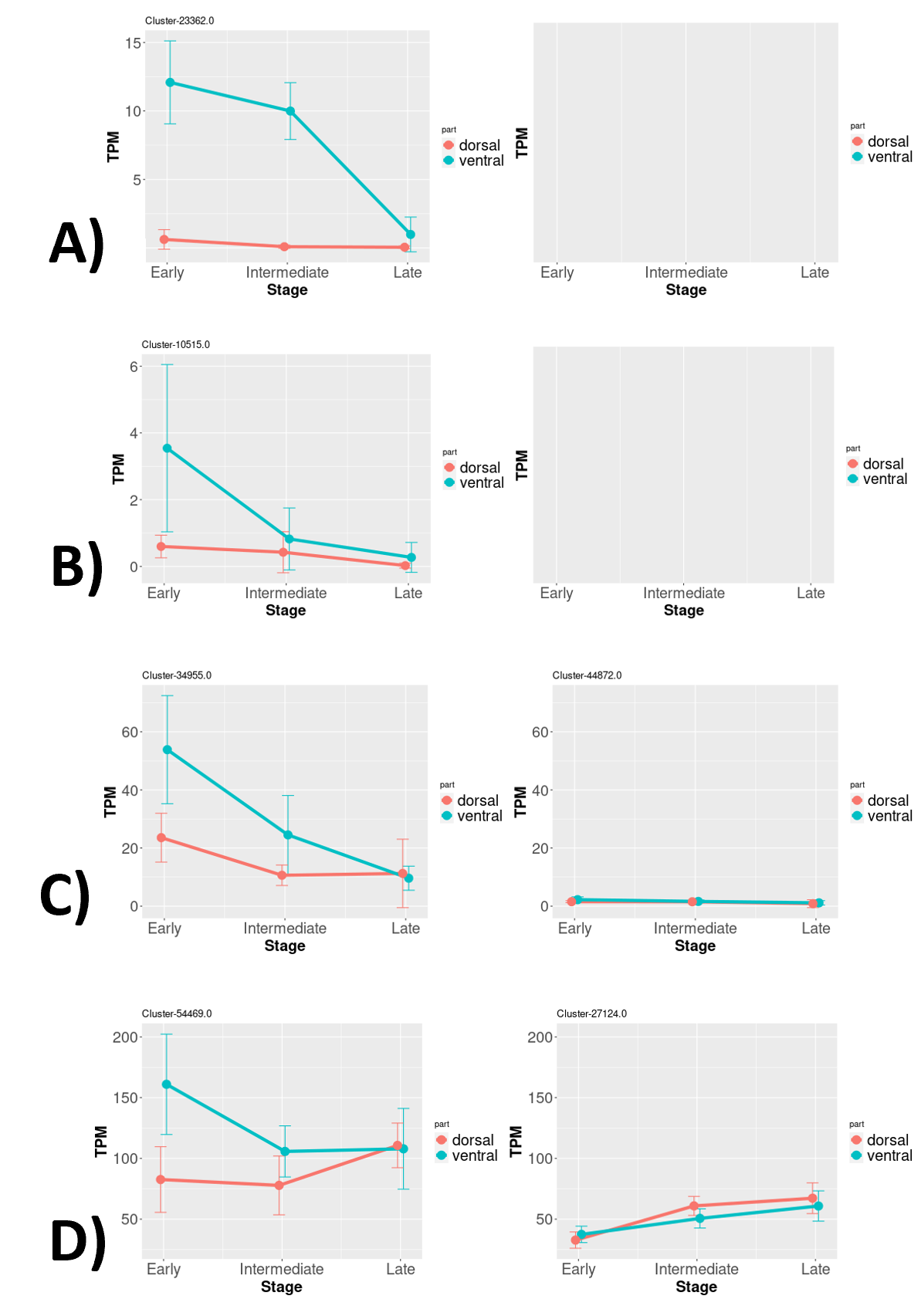


**Supplementary Figure 16.** Figure showing expression patterns of candidate genes. On the left hand side expression profiles of *L. vulgaris* are shown, on the right hand side expression of the ortholog in *A. majus* is shown. Bars indicate 95% confidence intervals. Annotation is from the top BlastX hit to Uniprot. (A) *FLORICAULA* (*FLO*). (B) *POPOVICH* (*POP*). (C) *TEOSINTE BRANCHED 1*/*CYCLOIDEA*/*PCF* (*TCP8*). (D) *IDD15* (*SHOOT GRAVITROPISM 5*).


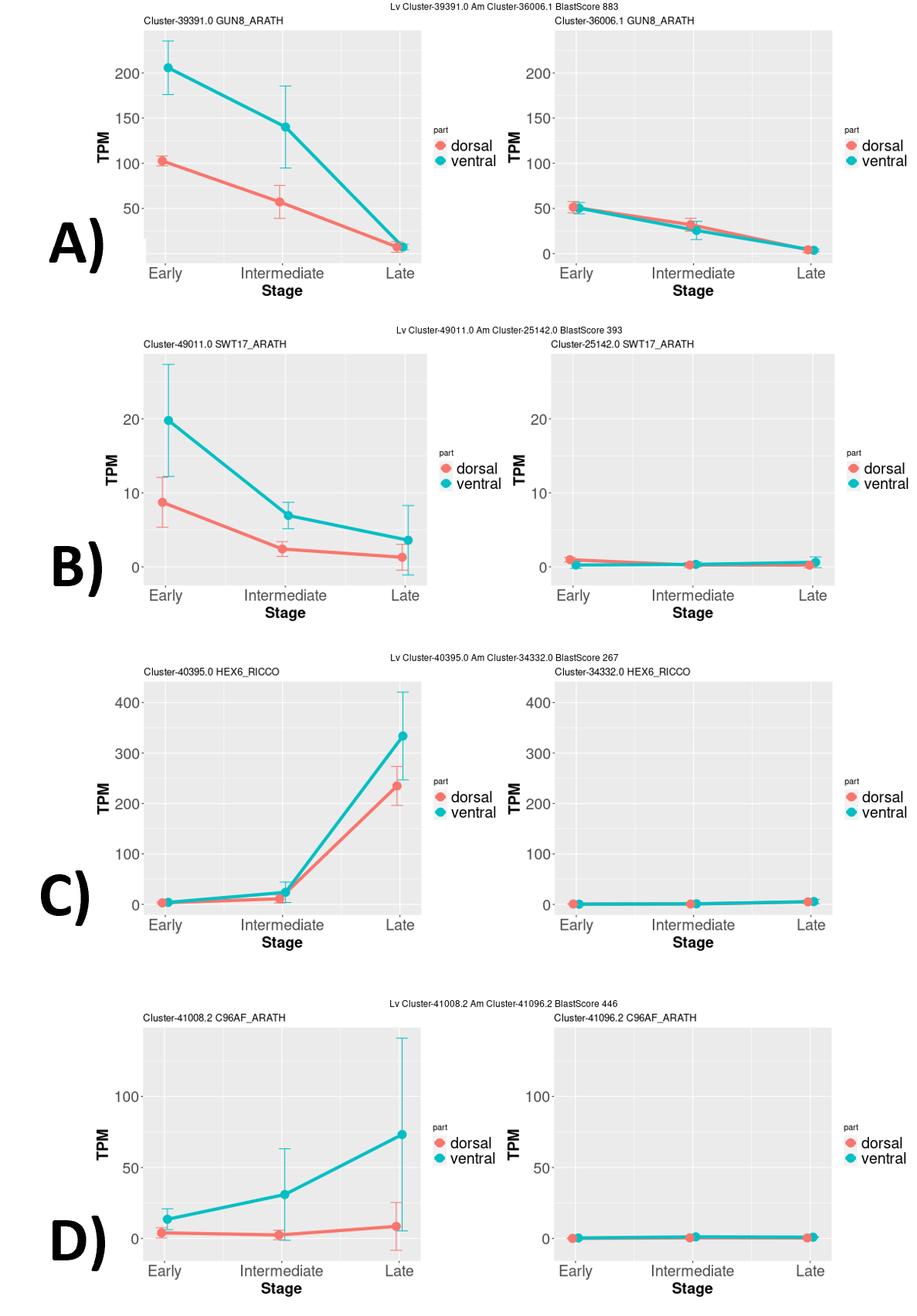


**Supplementary Figure 17.** Figure showing expression patterns of candidate genes. On the left hand side expression profiles of *L. vulgaris* are shown, on the right hand side expression of the ortholog in *A. majus* is shown. Bars indicate 95% confidence intervals. Annotation is from the top BlastX hit to Uniprot. (A) *ENDOGLUCANASE 8* (*GUN8*). (B) *SUGARS WILL EVENTUALLY BE EXPORTED TRANSPORTERS 17 (SWT17). (C) HEXOSE CARRIER PROTEIN HEX6* (*HEX6*). (D) *C96AF*.

## Supplementary Tables

**Supplementary table 1.** Table detailing the genes and gene names in each cluster in *Linaria vulgaris*. Uniprot ID, corset ID of gene in *L. vulgaris* de novo assembly, cluster ID and annotation is provided.

**Supplementary table 2.** Table detailing the genes and gene names in each cluster in *Antirrhinum majus*. Uniprot ID, corset ID of gene in *A. majus* de novo assembly, cluster ID and annotation is provided.

**Supplementary table 3.** Table decoding the gene names of spur aid genes (see heatmap, Figure 4). Uniprot ID, full gene name of nearest BLAST hit, and online link to BLAST hit is provided.

**Supplementary table 4.** Table decoding the gene names of spur suppressor genes (see heatmap, supplementary Figure 13). Uniprot ID, full gene name of nearest BLAST hit, and online link to BLAST hit is provided.
